# Early Multimodal Motor Training After Stroke Promotes Motor Recovery and Whole‐Brain Structural Remodeling

**DOI:** 10.1161/JAHA.124.039816

**Published:** 2026-07-03

**Authors:** Manuel Teichert, Sidra Gull, Karl‐Heinz Herrmann, Anja Urbach, Christiane Frahm, Ha‐Yeun Chung, Christian Gaser, Knut Holthoff, Jürgen R. Reichenbach, Otto W. Witte, Silvio Schmidt

**Affiliations:** ^1^ Department of Neurology Jena University Hospital Jena Germany; ^2^ Medical Physics Group, Institute of Diagnostics and Interventional Radiology Jena University Hospital Jena Germany; ^3^ Jena Center for Healthy Aging Jena University Hospital Jena Germany; ^4^ Section of Translational Neuroimmunology, Department of Neurology Jena University Hospital Jena Germany; ^5^ Department of Psychiatry and Psychotherapy Jena University Hospital Jena Germany; ^6^ Brain Imaging Center Jena Jena University Hospital Jena Germany

**Keywords:** brain remodeling, functional recovery, multimodal motor training, neurorehabilitation, structural magnetic resonance imaging, Ischemic Stroke, Cerebrovascular Disease/Stroke, Rehabilitation

## Abstract

**Background:**

Early task‐specific rehabilitation is critical for recovery after stroke, yet it remains unclear whether combining established rehabilitative training principles into an early multimodal motor training (EMT) paradigm improves recovery by engaging only local peri‐infarct adaptation or a broader, longitudinally detectable pattern of structural brain remodeling.

**Methods:**

Male rats were assigned to stroke+EMT, stroke, EMT, or control groups. Focal photothrombotic stroke was induced in the right sensorimotor cortex. EMT began 2 days after stroke and consisted of an obstacle‐based training paradigm with variable and progressively increasing difficulty delivered over 8 weeks. Skilled locomotion was assessed using the ladder‐rung test. Longitudinal T2‐weighted magnetic resonance imaging and deformation‐based morphometry were used to quantify structural brain alterations. Significant loci from voxel‐wise group×time interaction F‐contrast maps (family‐wise error‐corrected, *P*<0.05) were summarized as structural events and analyzed according to their spatial and temporal organization.

**Results:**

EMT improved poststroke motor recovery and was associated with broader structural remodeling than stroke or EMT alone (82 versus 15 and 10 structural events, respectively). These alterations extended beyond peri‐infarct tissue, were bilaterally distributed, and mapped to both sensorimotor and higher‐order brain systems, with frequent homotopic occurrence (74.4%) and multinetwork assignment (50.0%). Structural events were distributed across 6 temporal profiles, indicating distinct early‐to‐late remodeling courses rather than a single monotonic process.

**Conclusions:**

These findings support a shift in how early poststroke rehabilitation is conceptualized: not as a local process confined to isolated brain regions, but as a driver of distributed, bilateral, and temporally organized structural remodeling across multiple brain systems.

**Nonstandard Abbreviations and Acronyms**
AUD: auditory network category
CER: cerebellar network category
CPu: caudate‐putamen (striatum)
DBM: deformation‐based morphometry
DMN: default mode network
EMT: early multimodal motor training
EWEP: evidence‐weighted effect pattern clustering
FDR: false discovery rate
LIM: limbic network category
LR: ladder rung
ROI: region of interest
SAL: salience network category
SM: sensorimotor network category
STR: striatal network category
THAL: thalamic network category
VIS: visual network category

**Research Perspective**

**What Is New?**

- Early multimodal motor training after stroke improved motor recovery and was associated with a broader and more bilaterally distributed pattern of structural brain remodeling that extended beyond peri‐infarct tissue and involved both sensorimotor and higher‐order brain systems, with frequent homotopic occurrence and multinetwork assignment.
- Structural remodeling did not follow a single monotonic course, but instead showed distinct early‐to‐late temporal profiles across recovery.

**What Question Should Be Addressed Next?**

- Future studies should determine how these structural alterations relate to direct measures of circuit reorganization, including functional connectivity, tract‐level remodeling, and causal contributions to behavioral recovery.

Stroke remains a leading cause of mortality and long‐term disability worldwide.^1^, ^2^ Importantly, persistent contralateral motor impairments substantially limit independence and quality of life, underscoring the need for effective rehabilitative strategies.^3^, ^4^ Motor recovery occurs most prominently within the first weeks to months after stroke, when neuroplastic potential is highest before gradually declining.^5^, ^6^, ^7^, ^8^ This early phase is therefore considered a critical therapeutic window during which interventions can strongly influence later outcome.^9^, ^10^, ^11^

Consistent with this concept, early, task‐specific training of the impaired limb can partially restore function after stroke.^12^, ^13^, ^14^, ^15^ In both experimental and clinical neurorehabilitation, training principles, such as repetition, spacing, progressively increasing difficulty, variability, and multisensory stimulation, have each been associated with benefit.^16^

Recovery after stroke is increasingly understood as a distributed reorganization of functionally connected brain systems rather than a purely local peri‐infarct process. Clinical studies implicate not only sensorimotor networks, but also higher‐order systems, such as default mode, salience, attention, and frontoparietal networks, in poststroke impairment and recovery after motor stroke.^17^, ^18^, ^19^ Animal studies similarly describe a modular functional architecture that includes default‐mode–like, sensorimotor, striatal, and salience‐related systems.^20^, ^21^, ^22^ They further show that experimental stroke particularly disrupts interhemispheric sensorimotor connectivity, with partial restoration paralleling functional improvement.^23^ Together, these findings suggest that poststroke recovery relies on distributed functional reorganization across recovery‐relevant brain networks rather than being confined to lesion‐surrounding adaptation.

However, it remains unclear whether such functional effects of early rehabilitation after stroke are accompanied by structural brain remodeling. Previous work in healthy humans^24^, ^25^ and rodents^26^, ^27^ has shown that repeated training can induce measurable volumetric changes consistent with structural plasticity in task‐relevant brain areas. This raises the question of whether combining established rehabilitative training principles into an early multimodal motor training (EMT) paradigm after stroke can improve recovery and likewise elicit a longitudinally detectable structural remodeling response with a broader spatial organization.

In this rat study, we investigated whether EMT, applied during the acute to early subacute therapeutic window of heightened poststroke neuroplastic potential,^9^, ^11^ improves motor recovery and induces structural remodeling detectable by longitudinal magnetic resonance imaging (MRI). Using deformation‐based morphometry (DBM), we compared EMT‐related remodeling with the alterations observed after stroke or training alone and characterized its spatial and temporal organization across the poststroke brain.

## METHODS

### Transparency and Openness Promotion Statement

Data, methods used in the analysis, and study materials will be made available to any researcher for purposes of reproducing the results or replicating the procedure on reasonable request to the corresponding author.

### Animals

A total of 44 adult naïve male Wistar rats were included (RccHan:WIST, Charles River, Sulzfeld, Germany; 12 weeks old at baseline). Animals were housed in groups of 2 to 5 per cage under standard laboratory conditions (12‐hour light/dark cycle, temperature 21 °C ±1 °C, humidity 50%±10%) with ad libitum access to food and water. One week before baseline testing, rats were handled to habituate them to the experimenter and reduce procedure‐related stress. Health and behavior were monitored regularly throughout the study. No animals or data were excluded. All procedures followed the Animal Research: Reporting In Vivo Experiments (ARRIVE) guidelines.^28^ Every effort was made to minimize animal numbers and suffering. Experiments were conducted in accordance with German and European animal protection legislation and were approved by the local authority (Thüringer Landesamt für Lebensmittelsicherheit und Verbraucherschutz, Bad Langensalza, Germany).

### Study Design

**Figure 1:**
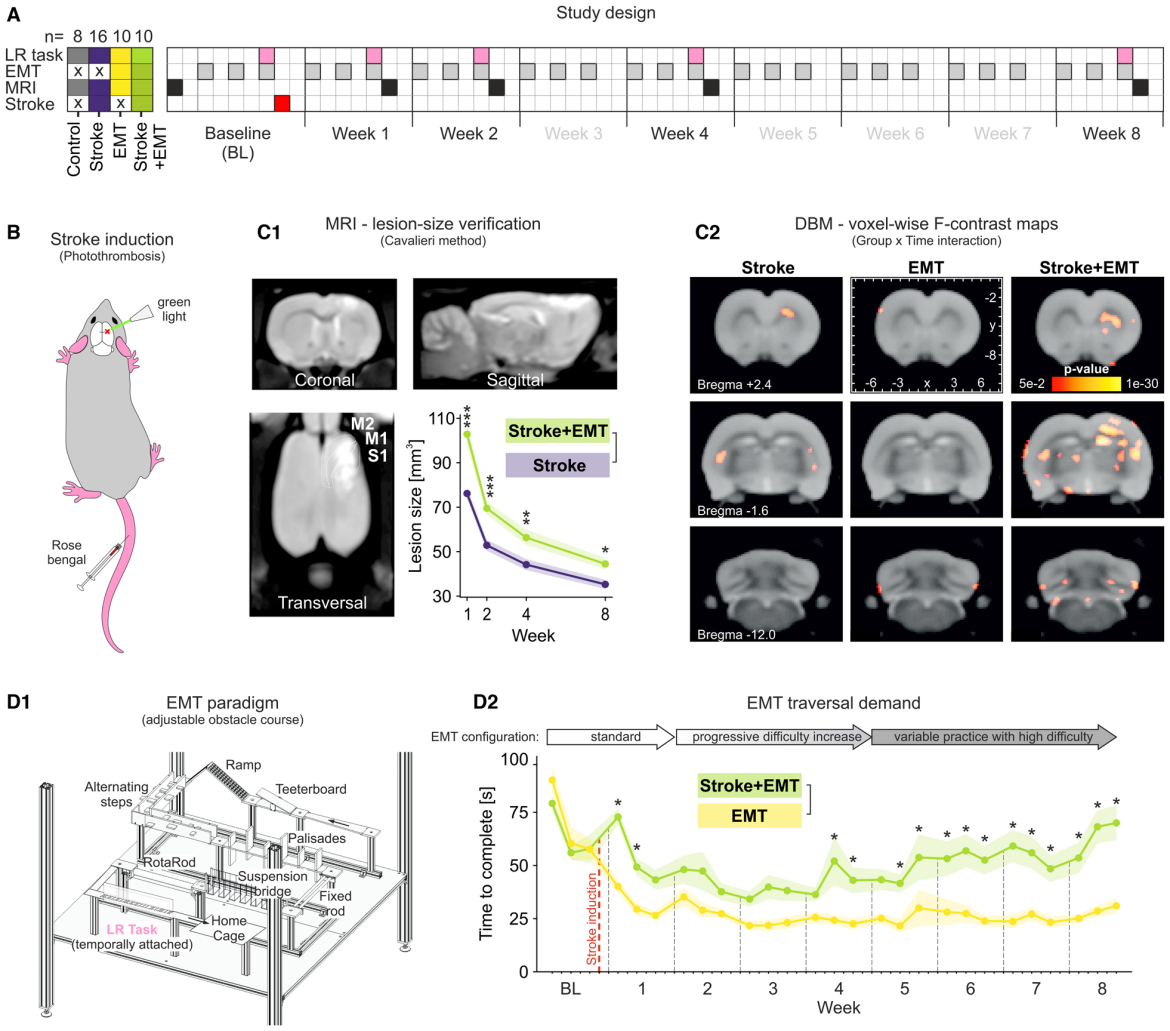
Study design, stroke induction, MRI‐based lesion characterization and F‐contrast maps, and early multimodal training. **A**, Left: Color code of the experimental groups. Filled squares indicate the presence and crosses indicate the absence of a specific intervention. Right: Timeline of the study design showing baseline assessments, stroke or sham surgery, EMT, LR testing, and MRI across the study period. **B**, Schematic of photothrombotic stroke induction targeting the right sensorimotor cortex. M2: secondary motor cortex; M1: primary motor cortex; and S1: primary somatosensory cortex. **C1**, Representative T2‐weighted MRI sections of a rat brain 8 weeks poststroke and longitudinal lesion‐size course in the stroke and stroke+EMT groups. Lesion sizes are shown as mean±SEM and were analyzed using a linear mixed‐effects model with group, time, and group‐by‐time interaction, followed by post hoc estimated marginal means contrasts at each time point. *P<0.05, **P<0.01, ***P<0.001. **C2**, DBM‐derived voxel‐wise group×time interaction F‐contrast maps illustrating localized structural alterations for stroke, EMT, and stroke+EMT vs controls, displayed as FWE‐corrected *P* values (flexible factorial repeated‐measures ANOVA, *P*<0.05) and projected onto the corresponding group‐mean MRI images. **D1**, Schematic of the EMT device with its adjustable obstacle elements. The LR segment at the end of the runway was attached only during behavioral assessment and was otherwise replaced by a neutral runway segment. **D2**, Times required to complete the EMT are shown as mean±SEM and were analyzed using 2‐way repeated‐measures ANOVA with factors group and time, followed by post hoc Tukey HSD, *P<0.05 for post hoc group differences. DBM indicates deformation‐based morphometry; EMT, early multimodal motor training; FWE, family‐wise error; HSD, honestly significant difference; LR, ladder rung; and MRI, magnetic resonance imaging.

This longitudinal controlled study examined how EMT after stroke affects motor performance and brain structure in adult rats. Animals were randomly assigned to 4 experimental groups (Figure 1A): control (no stroke, no training; n=8), EMT (no stroke, training; n=10), stroke (stroke, no training; n=16), and stroke+EMT (stroke, training; n=10). All animals underwent baseline assessments consisting of structural MRI on day −7 and the ladder rung (LR) test on day −1. On days −5, −3, and −1, animals assigned to the EMT and stroke+EMT groups were habituated to the training device in standard configuration. On day 0, focal ischemic stroke was induced in the stroke and stroke+EMT groups, whereas control and EMT animals underwent sham surgery. Starting on day 2, animals in the EMT and stroke+EMT groups performed EMT 3 times per week (Figure 1A). EMT was conducted independently of the LR test environment and contained no elements overlapping with behavioral testing. Follow‐up LR testing was performed in all groups and was paired with structural MRI on the following day. For clarity, longitudinal results are reported as week 1 (days 6/7), week 2 (days 13/14), week 4 (days 27/28), and week 8 (days 63/64). This design enabled longitudinal group‐wise comparisons of motor performance and structural brain alterations attributable to stroke, EMT, or their combination.

Group sizes ranged from 8 to 16 animals across the 4 experimental groups. The study was designed as a longitudinal mechanistic animal MRI experiment with repeated within‐subject measurements rather than around a single scalar primary end point. Group sizes were chosen to enable the 4‐group longitudinal design while limiting animal use in accordance with ethical reduction principles. Because all animals completed the study and underwent repeated behavioral and MRI measurements across the study period, the design increased within‐subject precision despite unequal group sizes.

### Stroke Induction

Focal cortical stroke was induced by photothrombotic occlusion targeting the right sensorimotor cortex^29^ (Figure 1B). Animals were anesthetized with isoflurane (2.0%–2.5% in a 70:30 mixture of nitrous oxide and oxygen) and received subcutaneous carprofen (5 mg/kg) for perioperative analgesia. Under sterile conditions, a polyvinyl catheter was inserted into the tail vein, the scalp was incised, and a fiberoptic light guide (aperture, 2.5 mm) was positioned 0.5 mm anterior to bregma and 3.7 mm lateral to the sutura sagittalis. During the first minute of cold green light illumination, rose bengal (1.3 mg/100 g body weight in 0.9% saline) was injected intravenously. Illumination continued for 20 minutes to induce local photochemical thrombosis of the cortical microvasculature. The catheter was then removed, the incision was sutured, and animals received pure oxygen until awakening and were monitored during recovery in a temperature‐controlled environment. Body temperature was monitored continuously using a rectal probe and maintained at 37 °C. All animals received carprofen (5 mg/kg, subcutaneously) once daily for 3 postoperative days. EMT and control animals underwent the same surgical procedures, but received sterile saline instead of rose bengal.

### Early Multimodal Motor Training

EMT was initiated 2 days after stroke or sham surgery and was performed 3 times per week for 8 weeks, with sessions spaced by 1 to 2 days. Each session consisted of 5 traversals of a 10‐cm wide runway elevated 40 cm above the ground and equipped with 7 obstacles designed to elicit complex motor behavior (Figure 1D1). The obstacle course targeted multiple components of motor control, including forelimb and hindlimb coordination, balance, adaptive gait, postural control, and sensorimotor integration. During the first week, animals were trained with the device in standard configuration. During weeks 2 to 4, obstacle difficulty was gradually increased (eg, by narrowing support surfaces or increasing instability), with modifications introduced after the first run. During weeks 5 to 8, the course was pseudorandomly reconfigured at each session at high difficulty to maintain continuous motor adaptation. Thus, EMT incorporated training principles considered relevant to poststroke motor recovery, including repetition within sessions, spaced practice across sessions, progressively increasing task difficulty, variable practice through obstacle reconfiguration, and multisensory stimulation through integrated visual, tactile, proprioceptive, and balance‐related demands.^16^ Times to complete the course were recorded as a surrogate measure of traversal demand, and were analyzed using 2‐way repeated‐measures ANOVA with factors group and time, followed by Tukey honestly significant difference post hoc tests.

### Obstacle Composition

The training device consisted of a course of 7 obstacles. The *teeterboard* was a seesaw that tilted under body weight and required anticipatory balance and postural adjustment, and was permanently in this standard configuration. The *ramp* combined a shallow ascent with a steep descent on a grid surface with oversized gaps and challenged propulsion, hindlimb use, and head‐down locomotion, and was permanently in this standard configuration. The a*lternating steps* formed a staircase with 12 steps of varying heights and distances, requiring adaptation to changing limb positions. In standard configuration, steps covered the entire width; difficulty was increased by gradually reducing paw‐placement surfaces and enlarging the gaps between steps, and variability by pseudorandom reconfiguration. The *palisade* consisted of 6 height‐adjustable vertical bars that required climbing in a narrow space with precise paw placement. In standard configuration, all bars were uniformly set to the lowest level; difficulty was increased by gradually alternating individual bar heights, thereby requiring repeated upward and downward climbing while maintaining stable limb placement, and variability by pseudorandom reconfiguration. The *parallel rods* consisted of 2 horizontal rods (5‐mm diameter) and challenged left–right coordination and balance. In standard configuration, rods were close together and firmly attached; difficulty was increased by gradually widening the distance between rods and loosening them to allow rotation, thereby requiring unfamiliar stepping strategies, and variability by pseudorandom reconfiguration. The *suspension bridge* consisted of 8 chain‐suspended steps. In standard configuration, the steps were stabilized by connecting wires and positioned at the same height; difficulty was increased by gradually removing the stabilizing wires to allow individual steps to swing, introducing height differences between steps, reducing step size, and replacing steps with rods suspended on only 2 chains, thereby increasing instability and balance demands, and variability by pseudorandom reconfiguration. The *rotarod* was a motorized rotating cylinder (5‐cm diameter) that required dynamic gait adjustment in real time. In standard configuration, the cylinder was stationary; difficulty was increased by accelerating rotation from 1 to 5 rpm, and variability by changing rotation direction and speed pseudorandomly. If an animal fell, the timer was paused and restarted after the animal was returned to the last position. Falls were rare across groups and were not analyzed further.

EMT was restricted to the EMT and stroke+EMT groups. The LR test was not part of the EMT intervention. On test days, the LR segment temporarily replaced a neutral runway section at the end of the parcours, ensuring strict separation between training and behavioral assessment.

### LR Test

**Figure 2:**
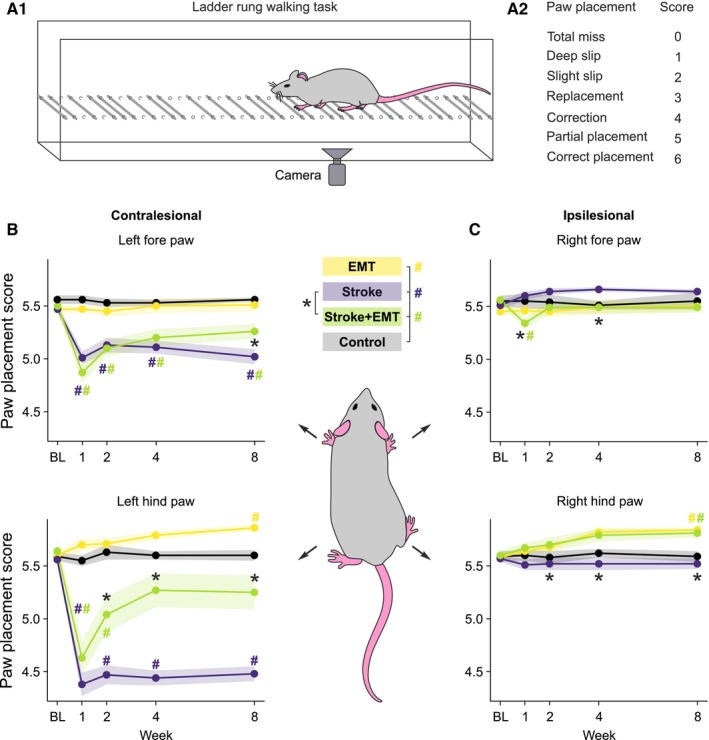
LR performance after stroke and early multimodal training. **A1**, Schematic of the LR walking task. **A2**, Paw‐placement scoring system used to quantify locomotor accuracy. **B**, Contralesional left forepaw and hindpaw placement scores over time. **C**, Ipsilesional right forepaw and hindpaw placement scores over time. Line plots show mean±SEM. Data were analyzed using 2‐way repeated‐measures ANOVA with factors group and time, followed by post hoc Tukey HSD. *P<0.05 for stroke vs stroke+EMT; #*P*<.05 for control vs EMT, stroke, or stroke+EMT. EMT indicates early multimodal motor training; HSD, honestly significant difference; and LR, ladder rung.

Motor performance was assessed using the LR test, a validated measure of locomotor coordination and paw‐placement accuracy (Figure 2A1). The apparatus consisted of a 1‐m horizontal ladder with irregular rung spacing (1–5 cm) and transparent Plexiglas side walls (19 cm high), requiring precise forelimb and hindlimb stepping. All animals performed 5 traversals per session at baseline and at follow‐up assessments in weeks 1, 2, 4, and 8. In the EMT and stroke+EMT groups, the ladder segment was temporarily attached to the training device on test days; in the remaining control and stroke groups, testing was performed separately. Locomotion was recorded using a high‐speed camera (100 frames/s; Pike F‐32) and analyzed frame by frame using the SIMI Motion software. Paw placement was scored on a 7‐point scale ranging from 0 (total miss) to 6 (correct full‐weight–bearing placement^30^; Figure 2A2). Average scores across the 5 traversals were calculated for each paw and time point. Scores were analyzed separately for each paw using 2‐way repeated‐measures ANOVA with factors group and time, followed by Tukey honestly significant difference post hoc tests. The LR test thus provided an independent readout of motor recovery and was not itself part of the training intervention.

### Magnetic Resonance Imaging

Structural MRI was performed longitudinally at 5 time points: baseline (day −7, before any intervention) and weeks 1, 2, 4, and 8 after intervention. Scans were acquired on a 3‐T whole‐body scanner (Magnetom TIM Trio, Siemens Medical Solutions, Erlangen, Germany) equipped with a custom rat head volume resonator (Doty Scientific Inc, Columbia, SC) using a linearly polarized Litz coil configuration. Animals were anesthetized with 1.7% isoflurane in oxygen (1.5 L/min) and positioned in the coil. Whole‐brain structural images were acquired using a high‐resolution 3‐dimensional T2‐weighted SPACE sequence with the following parameters: isotropic voxel size, 0.33 mm; matrix, 192×130×96; field of view, 64×43×32 mm; echo time, 352 ms; repetition time, 2500 ms; turbo factor, 67; echo spacing, 10.7 ms; bandwidth, 145 Hz/pixel; flip‐angle mode, T2var; and partial Fourier, 7/8 in the phase‐encoding directions. Each scan consisted of 2 repetitions, resulting in a total acquisition time of ≈14 minutes per animal. This protocol provided high‐resolution whole‐brain coverage suitable for voxel‐wise morphometric analysis.

### MRI Data Processing and Analyses

#### Step 1: Deformation‐Based Morphometry

Longitudinal structural alterations were quantified using DBM (SPM8, MATLAB R2018a^31^). For each animal, all postbaseline scans were nonlinearly registered to the individual baseline scan. Voxel‐wise Jacobian determinants were then computed to quantify local volume‐change values as percentage deviation from each animal's own baseline. The resulting DBM‐derived baseline‐referenced volume‐change maps were normalized to a study‐specific template described previously,^31^ which had been aligned to the Paxinos rat brain atlas space^32^ for anatomic assignment and between‐subject comparability. The resulting maps were spatially smoothed with a Gaussian kernel (full width at half maximum, 0.8 mm). This DBM framework served as the basis for all subsequent analyses of structural remodeling.

#### Step 2: Group‐Level Detection of Longitudinal Structural Remodeling Effects and Structural Event Definition

To identify brain locations showing group‐specific longitudinal structural remodeling effects, voxel‐wise group×time interactions were tested separately for stroke, EMT, and stroke+EMT versus controls using a flexible factorial repeated‐measures ANOVA. Resulting F‐contrast maps were thresholded at *P*<0.05, family‐wise error corrected, with a minimum cluster size of 10 voxels (Figure 1C2). Within each significant cluster, the voxel with the maximum F value was defined as the structural‐event peak and its atlas‐space location was treated as the corresponding structural‐event locus.

#### Step 3: Systems‐Level Mapping of Structural Events

To describe MRI‐derived data at a systems level, structural events were first assigned to anatomic areas according to the Paxinos rat brain atlas (*event‐to‐area assignment*). Multiple events within the same atlas area were retained as distinct subregional events. Hemisphere labels were defined relative to the lesion in the stroke groups (ipsilateral/contralateral), whereas in the EMT‐only group, hemispheres were described anatomically. Then, event‐bearing atlas areas were assigned to a study‐specific set of 9 literature‐guided network categories (*area‐to‐network assignment*): thalamic network category (THAL), sensorimotor network category (SM), cerebellar network category (CER), salience network category (SAL), default mode network (DMN), visual network category (VIS), limbic network category (LIM), auditory network category (AUD), and striatal network category (STR). Area‐to‐network assignments were derived from a manually curated literature corpus of animal network studies identified by semantic search and screened for explicit network‐level analysis, anatomic specificity, and relevance to rodent network organization or poststroke remodeling. Representative studies informing the systems‐level framework included work on bilateral resting‐state organization, default‐mode–like architecture, cross‐species network correspondence, triple‐network–like organization, and poststroke network remodeling.^20^, ^21^, ^22^, ^33^, ^34^, ^35^, ^36^ The full curated study corpus is provided in Table S1. Paxinos area abbreviations and network‐category abbreviations are summarized in the Table1. Because individual atlas areas were frequently described as a hub or assigned to multiple brain networks within the literature, area‐to‐network assignments were treated as nonexclusive, meaning that individual atlas areas could be assigned to multiple brain networks. Events without literature‐supported network assignment were retained as unassigned. From these initial assignments, we derived 5 descriptive summaries: (1) multinetwork assignment: applies to events whose atlas area was assigned to at least 2 networks, and therefore reflects events with *potential overlap across network* categories; (2) homotopic occurrence: applies to events with a corresponding event in the homotopic atlas area of the opposite hemisphere, and therefore *indicates the bilater*al representation of structural events across hemispheres; (3) event‐to‐network assignments: indicating how often structural events were assigned to the respective network, and therefore reflect how *strongly structural events wer*e represented across the different network categories; (4) network‐pair counts: indicating how often structural events were assigned to individual network pairs within the same hemisphere, and ther*e*fore describes the potential overlap structure of structural alterations between individual networks within the same hemisphere; and (5) network‐specific homotopy counts: indicating how often event‐bearing homotopic area pairs were assigned to the respective network, and therefore describes the *bilateral* representation of structural alterations within individual networks across hemispheres. Because area‐to‐network assignment was nonexclusive, the same structural event could contribute to multiple networks and multiple network pairs, and the same homotopic area pair could contribute to >1 network category. These summaries were used to describe event organization across known rodent brain systems and were not interpreted as direct measures of functional connectivity.

**Table 1:** Abbreviations of Paxinos Atlas Brain Areas and Network Categories

A	Anterior amygdaloid area	LS	Lateral septal nucleus
AI	Agranular insular cortex	LVPO	Lateroventral periolivary nucleus
AOL	Anterior olfactory nucleus, lateral part	M1	Primary motor cortex
APir	Amygdalopiriform transition area	M2	Secondary motor cortex
APTD	Anterior pretectal nucleus, dorsal part	PFl	Paraflocculus
Au1	Primary auditory cortex	Pir	Piriform cortex
AUD	Auditory network category	PLH	Peduncular part of lateral hypothalamus
AuV	Secondary auditory cortex, ventral area	Pn	Pontine nuclei
AVVL	Anteroventral thalamic nucleus, ventrolateral part	PRh	Perirhinal cortex
Cb2	Second cerebellar lobule	RS	Retrosplenial cortex
Cb2/3	Second and third cerebellar lobules	Rt	Reticular thalamic nucleus
Cb2b	2b Cerebellar lobule	S1BF	Primary somatosensory cortex, barrel field
Cb5	Fifth cerebellar lobule	S1FL	Primary somatosensory cortex, forelimb region
CER	Cerebellar network category	S1HL	Primary somatosensory cortex, hindlimb region
CPu	Caudate‐putamen (striatum)	S1J	Primary somatosensory cortex, jaw region
Crus	Crus of the ansiform lobule	S1ULp	Primary somatosensory cortex, upper lip region
DCDp	Dorsal cochlear nucleus, deep core	S2	Secondary somatosensory cortex
DCIC	Dorsal cortex of the inferior colliculus	SAL	Salience network category
DMN	Default mode network	SM	Sensorimotor network category
DS	Dorsal subiculum	STR	Striatal network category
Ect	Ectorhinal cortex	THAL	Thalamic network category
Ent	Entorhinal cortex	Tu1	Olfactory tubercle
FrA	Frontal association cortex	V1	Primary visual cortex
GrO	Granular cell layer of the olfactory bulb	V2L	Secondary visual cortex, lateral area
HC	Hippocampus	Ve	Vestibular nucleus
InG	Intermediate gray layer of the superior colliculus	VIS	Visual network category
LAcbSh	Lateral accumbens shell	VPM	Ventral posteromedial thalamic nucleus
LIM	Limbic network category	VS	Ventral subiculum
LPtA	Lateral parietal association cortex		

#### Step 4: Event‐Wise Longitudinal Inference and Temporal Classification

For each structural event, DBM‐derived baseline‐referenced volume‐change values were extracted from a 19‐voxel sphere centered on the event peak at all postbaseline time points (weeks 1, 2, 4, and 8). These event‐level trajectories were used to derive control‐referenced volume‐change effects and time point–specific statistical evidence. Control‐referenced volume‐change effects were obtained by subtracting the corresponding control mean volume‐change value at each time point from the values of each noncontrol group, yielding signed control‐referenced effects expressed in percentage points. Positive values indicate more positive volume change than in controls, whereas negative values indicate more negative volume change. To determine at which time points event‐level volume‐change trajectories differed from controls, each event was analyzed using repeated‐measures ANOVA followed by Tukey‐adjusted post hoc contrasts restricted to the prespecified comparisons of each noncontrol group versus controls at each time point. Resulting contrast‐specific *P* values were adjusted across events by the Benjamini‐Hochberg procedure separately within each group×time family, yielding false discovery rate (FDR)–adjusted q values. Statistical significance was defined as q<0.05.

To identify recurring longitudinal trajectory patterns across structural events, we applied evidence‐weighted effect pattern clustering (EWEP). Each event was represented by 2 feature blocks: a control‐referenced volume‐change effect block containing the 4 control‐referenced effects at weeks 1, 2, 4, and 8, and an evidence block capturing statistical support at the same time points. The effect block was standardized by column‐wise z scoring across events. Evidence scores were derived from FDR‐adjusted q values as signed quantities, defined as sign(control‐referenced effect)×min(−log10[q], 6) when q<0.05 and set to 0 otherwise, such that only statistically supported effects contributed evidence and extreme q values could not dominate the feature space. To preserve 0 as an explicit no‐evidence reference, the evidence block was scaled by SD only, without mean centering. The 2 blocks were then combined as X=[Z_effect, λ×Z_evidence], where λ controlled the relative weight of statistical evidence versus the control‐referenced effect block. K‐means clustering with Euclidean distance was performed on a predefined parameter grid (k=2–8; λ={0.5, 1, 2}). Model selection prioritized mean silhouette width, with mean adjusted Rand index across 40 random initializations used as tie‐breaker and robustness criterion. The final model was fit using the selected parameter combination with multiple random starts and a high iteration limit. The selected EWEP solution was the k=2, λ=0.5 model, which yielded the highest mean silhouette width among the evaluated parameter combinations and perfect stability relative to the reference solution (mean silhouette width 0.446; mean adjusted Rand index, 1.000).

For descriptive timing classification, each event was additionally assigned a temporal subclass based on FDR significance at week 1 and week 8: subclass a, significant at week 1 but not week 8; subclass b, significant at week 8 but not week 1; subclass c, significant at both week 1 and week 8; and subclass d, not significant at either week 1 or week 8. This temporal subtyping was descriptive and did not alter the EWEP cluster assignments. Event‐level volume‐change effects, FDR‐adjusted q values, temporal subclass assignments, and related characteristics are provided in Table S2.

#### Step 5: Distance‐to‐Stroke, Infarct‐Volume Dependence, and Squeezing‐Control Analyses

To separate spatial occurrence from longitudinal magnitude, we quantified for each event an early‐to‐late effect‐size span defined as the absolute difference between week 1 and week 8 effect‐size values. The relation between event location and this span was analyzed in 2 complementary ways. First, each event's Euclidean distance from the stroke center was assigned to predefined bins (0–3.5, 3.5–7, 7–10.5, 10.5–14, and 14–17.5 mm), and event counts were summarized across bins. Second, distance‐dependent scaling of effect‐size span was tested using unbinned Spearman correlations between each event's effect‐size span and its Euclidean distance from the stroke center. Because nearby events are not spatially independent, significance testing was based on spatially blocked permutation tests. Three‐dimensional coordinates were partitioned into 1‐mm spatial blocks, and empirical null distributions were generated from 10 000 permutations. Distance‐effect tests were performed separately for the 6 prespecified group×subclass combinations defined by stroke versus stroke+EMT and temporal subclasses a, b, and c, with Benjamini‐Hochberg correction across these 6 tests.

To test whether effect‐size spans were explained by lesion severity, we fitted a linear mixed‐effects model comparing stroke and stroke+EMT. Fixed effects were group, week 1 infarct volume, and their interaction; animal identifier was included as a random intercept. The dependent variable was the mean log10‐transformed effect‐size span between week 1 and week 8. To evaluate whether opposite‐signed effects could reflect a purely local mechanical squeezing artifact, we also performed spatial adjacency analyses in 3‐dimensional coordinate space using the same blocked‐permutation framework. Neighborhoods were defined either by fixed radii (2–5 mm) or by k‐nearest neighbors (k=1–5). At weeks 1 and 8, we quantified the fraction of opposite‐sign neighboring event pairs and tested local mixing of trajectory labels and temporal subclasses. Multiplicity across all group×time×neighborhood specifications was controlled using the Benjamini‐Hochberg procedure.

#### Step 6: ROI‐Level Regional Time‐Course Analysis

In addition to peak‐level structural‐event analyses, region‐level volumetric trajectories were analyzed to determine whether spatially integrated region of interest (ROI) signals mirrored or diverged from subregional peak‐level effects. ROI analyses were framed as prespecified group‐versus‐control contrasts at weeks 1 and 8 and were assessed separately for each ROI and hemisphere using linear mixed‐effects models on raw ROI volumes with animal identifier as a random intercept. Multiplicity was controlled using Benjamini‐Hochberg FDR correction across ROI×hemisphere tests within each contrast and time point. Based on FDR‐adjusted significance at weeks 1 and 8, each ROI was assigned a descriptive timing class analogous to the peak‐level temporal subtyping scheme.

### Lesion Volume Estimation

Lesion volume was quantified on T2‐weighted MRI at weeks 1, 2, 4, and 8 using the Cavalieri method.^37^ Coronal slices with a thickness of 0.33 mm were overlaid with a 1.0‐mm grid in ImageJ (National Institutes of Health). Hyperintense cortical regions were identified, and lesion volume was calculated as the sum of matching grid points multiplied by grid area and slice thickness. Analysis was performed blinded to group. Longitudinal lesion volumes were analyzed using a linear mixed‐effects model with fixed effects of group, time, and group‐by‐time interaction, a random intercept for animal, and an AR(1) within‐animal correlation structure. Post hoc group contrasts at individual time points were derived from estimated marginal means with multiplicity adjustment. Data are presented as mean±SEM.

### Statistical Analysis

Statistical procedures specific to each analysis are described in the corresponding Methods sections. Unless otherwise specified, statistical significance was defined as *P*<0.05 or q<0.05, as appropriate. Analyses were performed using MATLAB R2018a, IBM SPSS Statistics 29.0, and R.

## RESULTS

All animals completed the full study protocol (Figure 1A), including all 5 structural MRI examinations and the corresponding ladder rung assessments. Animals in the EMT and stroke+EMT groups additionally completed all 27 EMT sessions over the 8‐week study period. No dropouts or adverse events occurred, and all data were included in the analyses.

### EMT Traversal Demand Shows 2 Distinct Phases After Stroke

Both EMT and stroke+EMT rats successfully completed the EMT course across all training sessions. In EMT rats, the time required to complete the course decreased until week 3 and remained stable thereafter. Compared with EMT rats, stroke+EMT rats required significantly more time to complete the course in week 1, indicating higher traversal demand early after stroke. This was followed by a progressive reduction in course‐completion time until week 3. From week 4 onward, however, completion time in stroke+EMT rats gradually and significantly increased again until week 8, diverging from EMT rats during the phase of highest and variable obstacle‐course difficulty, suggesting that traversal demand increased again once course demands became both maximal and continuously changing (Figure 1D2).

### Stroke+EMT Rats Show Persistently Larger Lesion Volumes Than Stroke

Photothrombotic infarcts were centered in the right primary motor cortex and extended into adjacent parts of the secondary motor cortex and the forelimb and hindlimb regions of the primary somatosensory cortex (Figure 1C1 and Figure S1). Longitudinal lesion‐volume analysis using a linear mixed‐effects model showed significant effects of group, time, and group×time interaction. Post hoc estimated marginal means indicated larger lesion volumes in stroke+EMT than in stroke at weeks 1, 2, 4, and 8, whereas lesion‐volume means decreased over time in both groups.

### EMT Improves Poststroke Motor Recovery on the LR Task

We next assessed the effect of EMT on poststroke motor performance using the LR test as an independent behavioral readout of skilled locomotion. Healthy control rats showed stable, high paw‐placement accuracy across all 5 assessments, indicating that repeated LR testing did not alter task performance (Figure 2B and 2C). EMT rats performed largely similar to controls throughout follow‐up, with a slight improvement in hindpaw performance at week 8. In contrast, both stroke‐affected groups showed marked impairments of the contralesional forepaws and hindpaws at week 1 (Figure 2B). In stroke rats, these deficits persisted across all follow‐up time points. Compared with stroke, stroke+EMT rats improved progressively over time, and hindpaw scores approached control levels during the later follow‐up period. Ipsilateral right forepaw and hindpaw placement remained comparatively preserved overall, although modest group differences were also detectable at selected time points (Figure 2C). Together, these findings indicate that EMT facilitates substantial recovery of impaired motor performance and may promote the transfer of motor improvements to behaviors not explicitly trained during the intervention.

### Whole‐Brain Structural Remodeling After Stroke and EMT Is Distributed, Bilateral, and Organized Across Brain Systems

**Figure 3:**
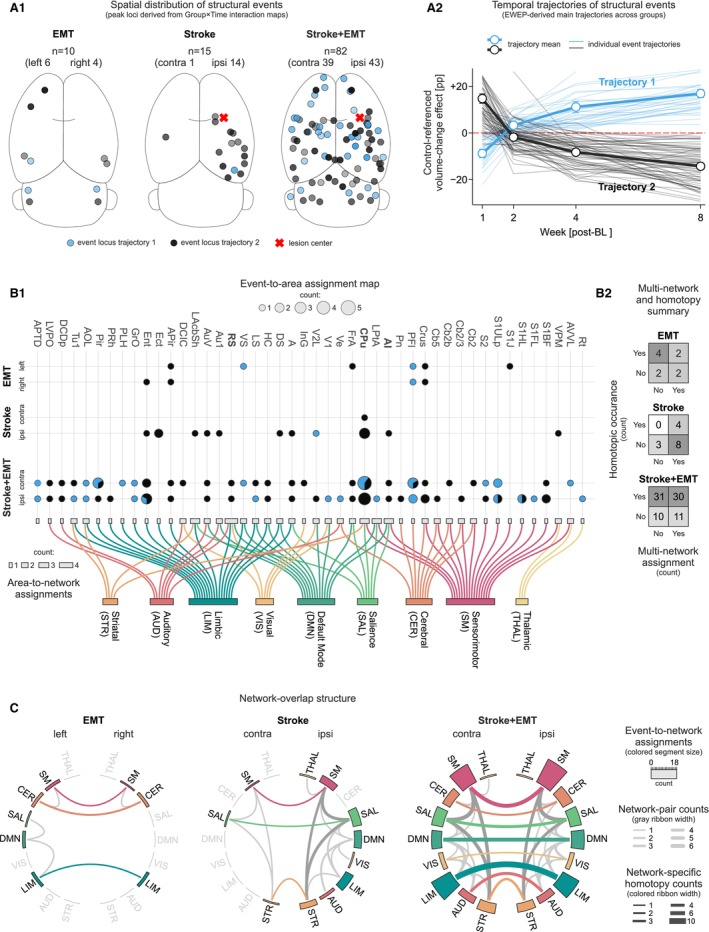
Spatial distribution, systems‐level mapping, and network‐overlap structure of structural events after stroke and early multimodal training. **A1**, Dorsal views of rat brains showing the spatial distribution of peak loci derived from voxel‐wise group×time interaction F‐contrast maps (Figure 1C2), defined as structural events and colored according to the 2 EWEP‐derived main trajectory types; darker and lighter shades indicate more dorsal or ventral events, respectively. Numbers above panels indicate total event counts; hemisphere‐specific counts are shown in parentheses. Red crosses denote the lesion center. **A2**, Temporal trajectories of structural events across groups. Thick lines represent mean±SEM of the 2 EWEP‐derived main trajectory types over time, and pooled across groups. Thin lines represent individual event trajectories. The y axis shows control‐referenced volume‐change effects (percentage points). **B1**, Event‐to‐area and area‐to‐network assignment map. Dot size indicates how often structural events were assigned to corresponding Paxinos atlas areas in each hemisphere. Colored ribbons indicate the assignment of atlas areas to a study‐specific set of brain‐network categories. Gray rectangle width indicates how many networks each area was assigned to. Paxinos area abbreviations are defined in Table1. **B2**, Multinetwork and homotopy summary. Individual structural events were cross‐classified according to whether their atlas area was assigned to at least 2 networks (multinetwork assignment) and whether a corresponding event was also present in the mirrored atlas area of the opposite hemisphere (homotopic occurrence). **C**, Chord diagrams summarizing the network‐overlap structure. Colored segment size indicates how often structural events were assigned to the respective network in the corresponding hemisphere (event‐to‐network assignments). Gray ribbon width indicates how often structural events were assigned to individual pairs of networks within the same hemisphere (network‐pair counts). Colored ribbon width indicates how often event‐bearing homotopic area pairs contributed to the respective network (network‐specific homotopy counts). Hemisphere‐resolved numeric counts underlying the chord diagrams are provided in Figure S2. Because area‐to‐network assignment was nonexclusive, the same structural event could contribute to multiple networks, network‐pair counts, and network‐specific homotopy counts. AUD indicates auditory; CER, cerebellar; DMN, default mode; EMT, early multimodal motor training; EWEP, evidence‐weighted effect pattern clustering; LIM, limbic; SAL, salience; SM, sensorimotor; STR, striatal; THAL, thalamic; and VIS, visual network category.

We next asked whether the behavioral improvement observed in stroke+EMT was accompanied by structural brain alterations detectable by longitudinal MRI and DBM. To capture localized group‐specific effects in a standardized way, we defined structural events as peak‐derived loci from voxel‐wise group×time interaction maps (Figure 1C2) and tracked their control‐referenced volume‐change effects across weeks 1, 2, 4, and 8. EWEP clustering resolved 2 main trajectories (Figure 3A2), which served as the global temporal frame of reference for the subsequent whole‐brain event analyses shown in Figure 3. Event‐level control‐referenced volume‐change effects, FDR‐adjusted q values, temporal subclass assignments, and related characteristics are provided in Table S2.

Stroke+EMT showed a markedly higher number of structural events than the other groups (Figure 3A1). In total, 82 events were detected in stroke+EMT, compared with 15 in stroke and 10 in EMT. Events in EMT were evenly distributed between hemispheres (6/10 left, 4/10 right), whereas events in stroke were predominantly localized to the hemisphere ipsilateral to the lesion (14/15). By contrast, events in stroke+EMT were distributed bilaterally (43/82 ipsilateral, 39/82 contralateral), indicating remodeling beyond strictly ipsilesional territories. Anatomic involvement was likewise broader in stroke+EMT, with events spanning 40 distinct atlas areas compared with 11 in stroke and 7 in EMT (Figure 3B1). Within stroke+EMT, the highest event counts were observed in the caudate‐putamen (CPu; 9 events), entorhinal cortex (5), and paraflocculus (5). Area abbreviations are defined in the Table1.

To place these anatomic findings into a systems‐level framework, event‐bearing atlas areas were further assigned to a study‐specific set of 9 literature‐guided network categories (Figure 3B1, for hemisphere‐specific counts see also colored segments in Figure 3C and the matrix in Figure S2). In EMT, assignments were sparse and concentrated mainly in cerebellar (CER; 4), limbic (LIM; 4), sensorimotor (SM; 3), DMN (2), and salience (SAL; 1) categories, with no assignments to thalamic (THAL), visual (VIS), auditory (AUD), or striatal (STR) systems. Stroke showed a broader but still limited pattern, with the highest counts in SAL (7), SM (6), LIM (6), DMN (5), and STR (5), together with isolated assignments to AUD (2), THAL (1), and VIS (1), whereas CER remained unrepresented. By contrast, stroke+EMT spanned all 9 network categories and showed the highest counts in SM (32), LIM (28), SAL (16), and DMN (16), followed by CER (14), STR (13), AUD (10), VIS (6), and THAL (2). In stroke+EMT, this profile was highly similar between hemispheres (Spearman ρ=0.91, *P*=0.0007), indicating strong ipsicontralateral concordance of event‐to‐network representation.

We next examined whether these events also differed in their interhemispheric and cross‐system organization. In EMT, 6 events showed homotopic occurrence, 4 showed multinetwork assignment, and 2 exhibited both properties (Figure 3B2). In stroke, multinetwork assignment was common (12 events) but remained largely confined to the ipsilesional hemisphere, with only 4 of these events also showing homotopic occurrence. Stroke+EMT showed the strongest bilateral and cross‐system organization, with 61 homotopic events and 41 multinetwork events, 30 of which combined both features. Thus, compared with stroke or EMT alone, stroke+EMT was characterized not only by more events, but also by a broader distribution across hemispheres and network categories.

A related pattern emerged from the overlap summaries in Figure 3C. EMT showed only sparse within‐hemisphere overlap between network categories. Stroke showed more pronounced overlap, but this remained largely ipsilesional and was centered on the combinations SAL‐SM (5), SAL‐STR (5), and SM‐STR (4). Stroke+EMT, in contrast, exhibited a broader and more balanced overlap structure. The most frequent network‐pair counts were SAL‐SM (12), SAL‐STR (9), and SM‐STR (9), followed by CER‐SM (7) and DMN‐LIM (7), all of which were distributed nearly symmetrically between hemispheres. A partly distinct pattern was seen for network‐specific homotopy counts: in EMT, homotopic event‐bearing area pairs were limited to CER (2), whereas in stroke they were sparse and restricted to SM (1), SAL (1), and STR (1). By contrast, stroke+EMT showed frequent homotopic representation, with the highest counts in LIM (10), SM (6), and DMN (6), followed by SAL (4) and AUD (4).

At the anatomic level, overlap in stroke was dominated by CPu events, accounting for 9 ipsilateral and 3 contralateral network‐pair counts and contributing mainly to an SAL‐SM‐STR motif that remained most prominent in the ipsilesional hemisphere. In stroke+EMT, CPu again dominated the overlap pattern, accounting for 15 contralateral and 12 ipsilateral network‐pair counts. Here, however, the same SAL‐SM‐STR motif was stronger overall and shifted toward a more balanced bilateral distribution. A secondary contribution arose from retrosplenial cortex, which accounted for 6 contralateral and 6 ipsilateral network‐pair counts, through combinations involving DMN, LIM, VIS, and AUD; and a third contribution arose from agranular insular cortex, which accounted for 3 contralateral and 3 ipsilateral network‐pair counts, through combinations involving SM, SAL, and DMN.

Taken together, stroke+EMT was associated with a substantially broader and more bilateral pattern of structural remodeling than stroke or EMT alone. At the systems level, this pattern was centered on a recurrent SAL‐SM‐STR motif, showed strong interhemispheric concordance, and was complemented by additional higher‐order involvement with frequent homotopic expression.

### EWEP Identifies 2 Main Trajectories and 6 Temporal Profiles With Distinct Systems‐Level Organization

**Figure 4:**
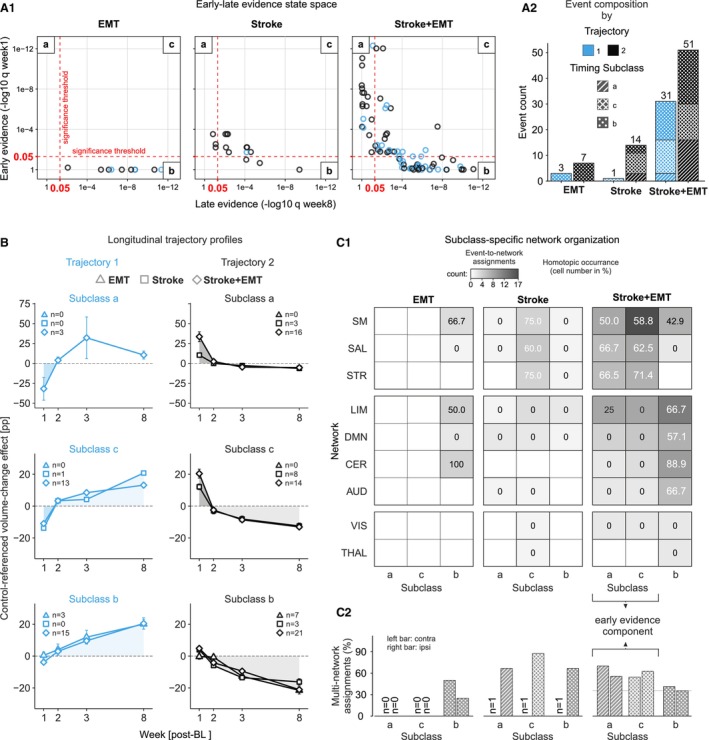
Temporal profiles and systems‐level organization of structural events after stroke and early multimodal training. **A1**, Early‐late evidence state space of structural events. Colors indicate the 2 EWEP‐derived main trajectories (k=2, λ=0.5; silhouette, 0.446; ARI, 1.000). Each point represents 1 structural event positioned by its early and late evidence at week 1 and week 8, displayed on a reversed logarithmic scale. Based on FDR significance at week 1 and week 8 (threshold corresponding to q=0.05, red dashed lines), structural events were additionally assigned to timing‐defined subclasses: subclass a, early‐transient; subclass b, late‐emergent; and subclass c, persistent‐evidence. No event was nonsignificant at both week 1 and week 8. **A2**, Stacked bars show the global composition of structural events across main trajectories, subclasses, and groups. Hemisphere‐resolved summaries are provided in Figure S3A. **B**, Longitudinal trajectories of the 6 temporal profiles. Control‐referenced volume‐change effects (percentage points) across follow‐up time points are shown as mean±SEM per group. **C1**, Subclass‐specific systems‐level organization, pooled across the 2 main trajectories. Cell colors indicate how often subclass‐specific structural events were assigned to the respective network category (event‐to‐network assignments). Cell labels indicate the percentage of subclass‐specific structural events assigned to the respective network category with a corresponding event in the mirrored atlas area of the opposite hemisphere (homotopic occurrence). Because area‐to‐network assignment was nonexclusive, the same structural event could contribute to multiple networks. Hemisphere‐resolved numeric counts underlying the heat maps are provided in Figure S3B. **C2**, Bars indicate the percentage of subclass‐specific structural events whose atlas area was assigned to at least 2 networks per hemisphere (structural events with multinetwork assignment). ARI indicates adjusted Rand index; AUD, auditory; CER, cerebellar; DMN, default mode; EMT, early multimodal motor training; EWEP, evidence‐weighted effect pattern clustering; LIM, limbic; SAL, salience; SM, sensorimotor; STR, striatal; THAL, thalamic; and VIS, visual network category.

Having characterized the spatial organization of structural events, we next examined their temporal profiles. Across groups, EWEP identified 2 main trajectories (k=2, λ=0.5; silhouette, 0.446; adjusted Rand index, 1.000): T1, characterized by an overall increasing trend, and T2, characterized by an overall decreasing trend (Figure 3A2). Events in stroke clustered almost exclusively into T2 (14/15). By contrast, events in both stroke+EMT and EMT were distributed across both main trajectories, with 31 stroke+EMT events in T1 and 51 in T2, and 3 EMT events in T1 and 7 in T2 (Figure 4A2).

To further describe timing, events were additionally classified in an early‐late evidence state space based on FDR‐adjusted significance relative to controls at week 1 and week 8 (Figure 4A1). This yielded 3 timing‐defined subclasses: subclass a (early‐transient; significant at week 1 but not week 8), subclass b (late‐emergent; not significant at week 1 but significant at week 8), and subclass c (persistent‐evidence; significant at both week 1 and week 8). No event fell into the double‐nonsignificant category. Combining the 2 main trajectories with these 3 timing‐defined subclasses yielded 6 temporal profiles (T1a, T1b, T1c, T2a, T2b, and T2c).

Temporal profile composition differed markedly across groups (Figure 4A2). EMT events were exclusively late‐emergent and distributed across both main trajectories (T1b=3, T2b=7). Stroke showed 4 temporal profiles and was enriched for persistent‐evidence events in T2 (T2a=3, T2b=3, T2c=8, and T1c=1). In contrast, stroke+EMT showed the broadest temporal spectrum, spanning all 6 temporal profiles, with late‐emergent events forming the largest component (T1a=3, T1b=15, T1c=13; T2a=16, T2b=21, T2c=14). In stroke+EMT, this broad temporal spectrum was similar between hemispheres (hemisphere×subclass χ^2^, *P*=0.675; Figure S3A).

The mean longitudinal effect profiles of these classes further highlighted their differing temporal organization (Figure 4B). Early‐transient profiles (T1a/T2a) showed pronounced early deviations from controls followed by attenuation toward later time points. Late‐emergent profiles (T1b/T2b) showed deviations that became apparent only at later follow‐up. Persistent‐evidence profiles (T1c/T2c), by contrast, remained significant at both week 1 and week 8 and were characterized by sustained but dynamically changing trajectories, including sign‐reversing courses in several groups.

We next examined whether these timing‐defined subclasses differed in their systems‐level organization. Overall, events containing an early evidence component were more closely aligned with the recurrent SAL‐SM‐STR motif, whereas late‐emergent events in stroke+EMT showed relatively stronger representation in DMN, LIM, CER, and AUD (cell colors in Figure 4C1). To describe this pattern, events were pooled across both main trajectories and summarized by subclass according to their event‐to‐network assignments. In EMT, subclass b was most prominent in CER and LIM (4 each), followed by SM (3), DMN (2), and SAL (1). In stroke, sparse subclass a events were distributed across DMN (2), together with single assignments in AUD, LIM, SAL, and SM, whereas subclass b remained similarly sparse. By contrast, subclass c in stroke showed a clear concentration within the SAL‐SM‐STR motif, with the highest counts in SAL (5), SM (4), and STR (4), together with additional assignments in LIM (3) and DMN (2) and single assignments in AUD, THAL, and VIS. A related but broader pattern emerged in stroke+EMT. Subclass a was most prominent in SM (8), SAL (6), STR (6), and LIM (8), whereas subclass c showed the highest counts in SM (17), SAL (8), STR (7), and LIM (8). By contrast, late‐emergent subclass b was most strongly represented in LIM (12), CER (9), SM (7), and DMN (7).

A related pattern was observed for homotopic occurrence (cell numbers in Figure 4C1). In stroke, homotopic events were restricted to subclass c, particularly in SM (3/4, 75.0%), STR (3/4, 75.0%), and SAL (3/5, 60.0%). In stroke+EMT, events containing an early evidence component again showed frequent homotopic occurrence within the SAL‐SM‐STR motif: subclass a showed the highest rates in SAL (4/6, 66.7%), STR (4/6, 66.7%), and SM (4/8, 50.0%), whereas subclass c showed the highest rates in STR (5/7, 71.4%), SAL (5/8, 62.5%), and SM (10/17, 58.8%). By contrast, subclass b showed stronger homotopic organization in CER (8/9, 88.9%), LIM (8/12, 66.7%), AUD (4/6, 66.7%), and DMN (4/7, 57.1%). Hemisphere‐resolved summaries are shown in Figure S3B.

A complementary view was provided by multinetwork assignments (Figure 4C2). In EMT, subclass b showed only infrequent multinetwork assignment. In stroke, multinetwork events were most prominent in subclass c in the ipsilateral hemisphere (87.5%, 7/8). In stroke+EMT, events containing an early evidence component were more frequently associated with multinetwork assignment than late‐emergent events in both hemispheres (subclass a: contralateral 70.0%, 7/10; ipsilateral 55.6%, 5/9; subclass c: contralateral 54.5%, 6/11; ipsilateral 62.5%, 10/16; subclass b: contralateral 41.2%, 7/17; ipsilateral 35.3%, 6/17).

Taken together, EMT broadened poststroke structural remodeling from a predominantly T2‐restricted pattern to a full spectrum of 6 temporal profiles. Within this broader temporal repertoire, events containing an early evidence component remained preferentially linked to the recurrent SAL‐SM‐STR motif, whereas later‐emergent events contributed relatively more to additional higher‐order brain systems.

### Control Analyses Support a Biologically Organized Remodeling Pattern

Additional analyses were performed to further characterize the spatial determinants and scale dependence of the observed remodeling pattern. We first asked whether timing‐defined subclasses differed systematically in their spatial relation to the lesion (Figure S4). Across both stroke‐affected groups, events from subclasses a, b, and c were represented across all predefined lesion‐distance bins (0–3.5, 3.5–7, 7–10.5, 10.5–14, and 14–17.5 mm), indicating that subclass‐specific remodeling was not confined to a narrow perilesional shell but extended from near‐lesion to more distant locations.

We next tested whether the magnitude of structural alterations scaled with lesion distance by quantifying, for each event, the early‐to‐late effect‐size span, defined as the absolute difference between its week 1 and week 8 effect‐size values (Figure S4). Overall, effect‐size spans showed no clear concentration within specific lesion‐distance bins. Only early‐transient events (subclass a) in stroke+EMT showed a modest tendency toward larger spans closer to the lesion (ρ=−0.52, *P*=0.0283, q=0.17), whereas associations for stroke+EMT subclasses b and c were weaker and likewise did not survive correction for multiple testing (subclass b: ρ=−0.27, *P*=0.114, q=0.228; subclass c: ρ=−0.33, *P*=0.0971, q=0.228). Thus, lesion‐distance dependence was limited in the present data set.

We then asked whether the broader remodeling pattern in stroke+EMT could be explained by initial lesion size. Using mean log10‐transformed effect‐size span as the outcome, a mixed‐effects model including group, week 1 infarct volume, and their interaction detected no main effect of infarct volume (*P*=0.921), no group×lesion‐size interaction (*P*=0.748), and no residual group effect after accounting for lesion size (*P*=0.740). These findings indicate that the broader remodeling pattern in stroke+EMT was not explained by initial lesion size alone.

Finally, we tested whether opposite‐signed structural events could reflect a purely local mechanical squeezing artifact (ie, whether swelling in 1 region might simply displace adjacent tissue and thereby produce apparent shrinkage nearby, or vice versa). Spatial adjacency analyses in 3‐dimensional coordinate space showed no enrichment of opposite‐sign neighboring event pairs. In stroke+EMT, this was evident in the radius‐based analysis at 5 mm, where the opposite‐sign fraction was 0.449 at week 1 and 0.466 at week 8, and was similarly seen in the k‐nearest‐neighbor sensitivity analysis (k=5: 0.456 at week 1 and 0.468 at week 8). Likewise, local mixing of trajectory labels and timing‐defined subclasses showed no detectable nonrandom organization. These findings were consistent across all tested radii and k‐nearest‐neighbor specifications.

Together, these control analyses argue against simple lesion‐distance, lesion‐size, or local tissue–displacement accounts and instead support a biologically organized remodeling pattern.

### ROI‐Level Trajectories Complement Rather Than Mirror Peak‐Level Event Dynamics

Finally, we asked whether region‐level volumetric trajectories mirrored the dynamics of the peak‐derived structural events they contained (Figure S5). ROI analyses were performed for selected areas containing multiple events in stroke+EMT, including CPu, entorhinal cortex, and piriform cortex, and additionally for contralesional motor cortices (primary motor cortex and secondary motor cortex). Across regions, ROI‐level trajectories did not consistently follow the directionality of their embedded peak‐level events. In stroke, the contralateral CPu showed no ROI‐level alteration despite pronounced dynamics in its subregional peak‐derived events, whereas the ipsilateral CPu showed an early ROI‐level volume reduction that remained present at week 8. A similar dissociation was observed in stroke+EMT. Although entorhinal cortex contained multiple peak‐derived structural events, ROI‐level analysis showed no net change at either week 1 or week 8, consistent with cancellation of spatially heterogeneous subregional effects when averaged across the whole region. By contrast, piriform cortex showed a bilateral transient ROI‐level volume reduction at week 1 despite mixed peak‐level directions within the same anatomic area. Conversely, ROI‐level effects could also emerge in the absence of corresponding peak‐derived events: contralesional primary motor cortex and secondary motor cortex showed significant volume reductions at week 8 in both stroke and stroke+EMT despite the absence of corresponding peak‐derived events. Together, these findings indicate that peak‐level analyses captured localized, trajectory‐resolved remodeling foci, whereas ROI‐level analyses reflected broader net volume shifts across predefined anatomic territories.

## DISCUSSION

The present study shows that EMT improves recovery after cortical stroke in rats and, compared with the untrained stroke group, is associated with a broader, more bilateral, and more temporally diverse pattern of structural brain remodeling.

The finding that EMT improved LR performance after stroke, despite the task not being part of the training itself, argues against a simple familiarity effect and instead suggests broader improvement in motor adaptability. Such cross‐task transfer is consistent with the practice structure of EMT, which imposes multisensory demands on balance, coordination, and postural control.^16^ Comparable benefits have been reported in rodents after enriched or behaviorally complex training paradigms, particularly during the early subacute phase of increased plasticity after stroke.^14^, ^15^, ^38^ Notably, the improvement in LR performance occurred in parallel with a biphasic change in EMT course completion time, characterized by initially elevated traversal demand after stroke, partial adaptation, and a renewed increase once the obstacle course reached its highest and most variable difficulty. Together, this pattern aligns with rehabilitation concepts emphasizing early, intensive, and behaviorally rich training as an important determinant of poststroke recovery.^10^, ^11^

A central implication of the present data is that EMT‐related structural brain remodeling is best interpreted within a systems‐level framework rather than as a purely perilesional response. Clinical studies increasingly suggest that recovery depends not only on local repair,^17^, ^18^, ^39^ but also on reorganization across sensorimotor and higher‐order networks.^40^, ^41^ In line with this view, stroke+EMT rats showed structural alterations extending across multiple brain systems, including sensorimotor, salience, striatal, limbic, default mode, and cerebellar components, indicating that EMT‐related remodeling was not restricted to motor territories. Importantly, this pattern was not anatomically diffuse. Instead, the overlap structure pointed to a recurrent SAL‐SM‐STR motif, driven largely by caudate putamen events, together with additional contributions from retrosplenial and insular cortices that linked higher‐order systems with primary sensory or somatosensory components. Taken together, these features are consistent with a bilaterally distributed recovery‐associated remodeling pattern rather than a purely unilateral or lesion‐surrounding response.

Rodent functional imaging studies provide an important bridge for this interpretation by describing a modular brain architecture that includes default mode–like, sensorimotor, salience‐like, striatal, and other networks, with substantial parallels to human large‐scale organization.^20^, ^21^, ^34^, ^42^ More recent work has also supported triple‐network–like organization in rodents involving default mode–, salience‐, and executive‐like systems.^22^ Within this framework, our MRI‐based morphometric approach did not constitute a direct measurement of functional connectivity, but it does provide a biologically informed basis for interpreting why EMT‐related structural events clustered in network‐relevant territories rather than appearing anatomically arbitrary.

The temporal analyses add a further layer of interpretation. EMT not only broadened the spatial pattern of structural events, but also expanded their organization across 2 main trajectories and 6 temporal profiles. Events with an early evidence component were more often associated with overlap among salience, sensorimotor, and striatal network components, whereas late‐emergent events more often involved limbic, default mode, and cerebellar components. Clinical functional imaging studies likewise indicate that recovery‐related network changes are temporally heterogeneous, with some systems showing early disturbance and partial renormalization and others emerging or reconfiguring later across subacute and chronic phases after stroke.^40^, ^41^, ^43^, ^44^ Our findings extend this principle to the structural domain.

How should these temporal profiles be interpreted biologically? One plausible interpretation is that at least part of the positive‐to‐negative course within the identified temporal profiles reflects an expansion‐to‐renormalization process, in which early positive effects are followed by later attenuation or reversal as reorganization stabilizes.^24^, ^45^ By contrast, part of the later‐evolving positive effects observed in stroke+EMT rats may reflect delayed training‐related recruitment as task demands increased over time. However, these temporal profiles should not be interpreted as direct markers of any single cellular process. Glial responses, inflammatory dynamics, scar maturation, synaptic and dendritic remodeling, vascular changes, and atrophy‐related processes may all contribute to MRI‐based morphometric effects.^9^, ^46^, ^47^, ^48^, ^49^ The spatiotemporal profiles shown here are therefore best regarded as macroscopic signatures of brain tissue remodeling rather than direct readouts of specific biological mechanisms.

Additional analyses indicated that the observed patterns were not simply explained by lesion distance, lesion size, or local geometric effects. Lesion size was nevertheless consistently larger in stroke+EMT rats throughout follow‐up. One possible explanation is that early behavioral demand may interact with perilesional tissue vulnerability and subsequent repair processes, including scar maturation, as suggested in experimental animal studies after stroke.^46^, ^50^, ^51^, ^52^, ^53^, ^54^ Our findings do not by themselves indicate a net harmful effect of EMT, because stroke+EMT rats nevertheless showed improved motor recovery together with a broader and more bilaterally organized remodeling pattern that was not explained solely by initial lesion size.

Several limitations should be considered. First, the photothrombotic model yields reproducible cortical infarcts but does not capture the full heterogeneity of human stroke, particularly with respect to reperfusion dynamics, lesion topology, and deep subcortical involvement. Second, the systems‐level framework used here is descriptive and based on area‐to‐network assignments derived from a manually curated corpus of rodent network studies. Accordingly, the present analysis does not measure functional connectivity directly, and species differences in network architecture warrant caution in translational interpretation. Third, MRI‐based morphometric analyses provide indirect morphologic proxies of remodeling, whose interpretation depends on the chosen reference, and do not by themselves establish causal functional roles. Integration with functional imaging, tract tracing, diffusion‐based connectivity, electrophysiology, or targeted perturbation approaches will be necessary to determine how the identified structural events relate to network‐level reorganization and behavior. More dedicated approaches could help to resolve whether the macroscopic alterations observed here map onto defined intrahemispheric and interhemispheric corticocortical architectures, interareal inhibitory motifs, and activity signatures of functionally characterized neurons.^55^, ^56^, ^57^

Taken together, the present findings support a shift in how early poststroke rehabilitation is conceptualized: not as a local process confined to isolated brain regions, but as a driver of distributed, bilateral, and temporally organized structural remodeling across brain systems. This perspective aligns rehabilitation with contemporary models of recovery as a distributed network process and suggests that the effectiveness of restorative interventions may depend on their capacity to engage broad network reorganization.

## Sources of Funding

This work was supported by the European Union's Horizon 2020 research and innovation program (Marie Sklodowska‐Curie grant, SmartAge, grant 859 890) to O.W.W., C.G., and C.F.; the Carl Zeiss Foundation (IMPULS, grant P2019‐01‐006) to C.F. and C.G.; the German Research Foundation (OrganAge WI 830/12‐1, WI 830/12‐2, grant 413 668 513) to O.W.W. and Excellence Cluster “Precision Medicine in Chronic Inflammation” (grant EXC2167) to C.F.; the Federal Ministry of Education and Research (ERA PerMed, Pattern‐Cog, grant ERAPERMED2021‐127) to C.G., Bernstein Focus (grant 01EO1002) to S.S., and Jena Centre for Systems Biology of Aging, JenAge (grant 0315581) to C.F. and O.W.W.; the Interdisciplinary Center of Clinical Research of the Medical Faculty Jena (grants AMSP06, MSP12, and AMSP11) to A.U., S.G., and M.T.; the Else Kröner‐Fresenius Foundation (JenaSchool for Ageing Medicine and Else Kröner Research School, AntiAge) to O.W.W.; and the Center for Sepsis Control and Care (project STARDUsT) to H.Y.C.

## Disclosures

The authors declare no competing interest.

## Supporting information

Tables S1–S2
Figures S1–S5
References: [58–88]
ARRIVE checklist

ARRIVE checklist
