# Supplementary material for: Early Multimodal Motor Training After Stroke Promotes Motor Recovery and Whole‐Brain Structural Remodeling
Source: J Am Heart Assoc. 2026 Jul 3;15(13):e039816. doi: 10.1161/JAHA.124.039816 (PMC13477302; doi:10.1161/JAHA.124.039816)
Supplement: Supplementary file 2 [file JAH3-15-e039816-s002.pdf]

Supplemental Material

Table S1. Curated literature corpus used for area-to-network assignments.

Studies included in the manually curated corpus are shown together with their study focus/role and the network categories informing assignments.
Network abbreviations: SM, sensorimotor; DMN, default mode; VIS, visual; THAL, thalamic; LIM, limbic; STR, striatal; SAL, salience; AUD, auditory; CER, cerebellar.

Study (short citation)	Study focus / role in corpus	Networks informing assignments
Sierakowiak et al., 2015 [20]	Global network architecture	SM, DMN, THAL, LIM, STR
Gozzi and Schwarz, 2016 [21]	Review / network framework	SM, DMN, VIS, STR, SAL, AUD, CER
Green et al., 2018 [23]	Stroke / recovery	SM, THAL, STR
Jonckers et al., 2011 [33]	Global network architecture	SM, DMN, VIS, LIM, STR, AUD
Lu et al., 2012 [34]	DMN-focused network definition	SM, DMN, VIS, AUD
van Meer et al., 2010 [35]	Stroke / recovery	SM, DMN, THAL
Grandjean et al., 2019 [36]	Global network architecture	SM, DMN, VIS, THAL, LIM, STR, SAL, AUD, CER
Ash et al., 2016 [58]	Aging / baseline variation	DMN, LIM, SAL
Bardella et al., 2016 [59]	Global network architecture	SM, DMN, VIS, THAL, LIM, STR, SAL
Becerra et al., 2011 [60]	Global network architecture	SM, DMN, THAL, LIM, SAL, CER
Brier et al., 2022 [61]	Inflammation / perturbation	SM, DMN, SAL
Chuang and Nasrallah, 2017 [62]	Review / network framework	SM, DMN, VIS, LIM, STR, SAL
D'Souza et al., 2014 [63]	Global network architecture	SM, DMN, VIS, LIM, STR
Egimendia et al., 2019 [64]	Aging / baseline variation	SM, DMN, THAL, LIM
Ferrier et al., 2020 [65]	DMN perturbation / functional validation	SM, DMN, THAL, STR
Hakon et al., 2017 [66]	Stroke / recovery	SM, VIS, LIM, AUD
Hsu et al., 2016 [67]	DMN-focused network definition	SM, DMN, VIS, AUD
Hutchison et al., 2010 [68]	Global network architecture	SM, DMN, VIS
Ji et al., 2018 [69]	DMN perturbation / behavior	DMN, LIM, SAL
Kalthoff et al., 2013 [70]	Methods / anesthesia effects	SM, DMN
Lee et al., 2021 [71]	Methods / pipeline resource	SM, DMN, VIS
Liang et al., 2013 [72]	THAL-focused network definition	SM, VIS, THAL
Liang et al., 2015 [73]	Global network dynamics	SM, DMN, VIS, THAL, LIM, STR
Liska et al., 2015 [74]	Global network architecture	SM, DMN, STR, SAL
Liu et al., 2022 [75]	Inflammation / perturbation	DMN, LIM, AUD
Mandino et al., 2021 [76]	Triple-network / salience framework	SM, DMN, SAL
Mechling et al., 2014 [77]	Global network architecture	SM, DMN, VIS, STR
Minassian et al., 2020 [78]	Stroke / recovery	SM, THAL, STR
Nasseef, 2015 [79]	Methods / directed connectivity	SM, DMN, VIS
Otte et al., 2014 [80]	Network perturbation / methods	SM, DMN, LIM
Schwarz et al., 2013 [81]	Global network architecture	SM, DMN
Sforazzini et al., 2014 [82]	Global network architecture	SM, DMN, VIS, THAL, STR, AUD, CER
Straathof et al., 2019 [83]	Structure-function mapping	SM, DMN, VIS, THAL
Tu et al., 2020 [84]	DMN perturbation / salience framework	DMN, THAL, SAL
van Meer et al., 2012 [85]	Stroke / recovery	SM, DMN, THAL
Zhang et al., 2010 [86]	Global network architecture	SM, DMN, VIS
Zhu et al., 2019 [87]	Inflammation / perturbation	DMN, LIM, STR, SAL, AUD
Özalay et al., 2024 [88]	Aging / baseline variation	SM, DMN, THAL, LIM

Table S2. Event-level characteristics, time point-specific control-referenced volume-change effects, FDR-adjusted q values, and corresponding baseline-referenced Control and Group volume-change values of structural events. Event-level characteristics include structural event identifier, group, anatomical area, hemisphere, trajectory profile, and cluster-level F value. Time point-specific values are provided for Weeks 1, 2, 4, and 8 and include control-referenced volume-change effects (percentage points), FDR-adjusted q values, and the corresponding mean baseline-referenced Control and Group volume-change values (%). Control-referenced volume-change effects were derived by subtracting the corresponding Control mean at each time point. Trajectory profiles combine the 2 EWEP-derived main trajectories with the timing-defined subclasses. Temporal subclassification was based on FDR-adjusted significance at Week 1 and Week 8. Area abbreviations are defined in Table 1.

Group	Event	Area	Hemisphere	Profile	Effect size Week				F value	q-value Week				Volume change Control Week				Volume change Group Week			
					1	2	4	8		1	2	4	8	1	2	4	8	1	2	4	8
EMT	1	APir	ipsi	2b	-5 ± 0.9	-4.9 ± 0.6	-12.6 ± 1.2	-23.6 ± 1.5	25.77	1.00	1.00	4.40e-04	2.16e-11	8.2 ± 2.5	8.2 ± 2.4	14.6 ± 2.4	25.4 ± 1.9	3.1 ± 0.9	3.3 ± 0.6	2.1 ± 1.2	1.8 ± 1.5
EMT	2	APir	contra	2b	-4.5 ± 1.1	-5.2 ± 1	-11.8 ± 1.5	-23.9 ± 1.2	17.20	1.00	1.00	0.0078	4.43e-09	4.8 ± 1.8	4.3 ± 2.1	9.4 ± 3.3	18.4 ± 4.3	0.3 ± 1.1	-0.9 ± 1	-2.4 ± 1.5	-5.5 ± 1.2
EMT	3	Crus	ipsi	2b	-2.9 ± 1.4	3.7 ± 1.4	-5.8 ± 1.4	-20.2 ± 1.5	20.24	1.00	1.00	0.27	3.68e-08	6.9 ± 4	-1.5 ± 2.2	3.9 ± 3.1	21 ± 3.3	4 ± 1.4	2.2 ± 1.4	-1.9 ± 1.4	0.8 ± 1.5
EMT	4	Crus	contra	2b	-0.4 ± 2	1 ± 2.8	-12.3 ± 2.3	-21.8 ± 1.6	17.80	1.00	1.00	0.0069	7.27e-07	3.3 ± 2.1	1.5 ± 3.1	7.6 ± 3.8	21.9 ± 4.2	2.9 ± 2	2.5 ± 2.8	-4.7 ± 2.3	0.1 ± 1.6
EMT	5	Ent	ipsi	2b	-0.7 ± 1.1	0 ± 2.2	-13.3 ± 1.8	-23.5 ± 2.2	30.20	1.00	1.00	6.62e-04	1.80e-09	7.5 ± 2.4	9.4 ± 1.4	21.6 ± 2.6	34.9 ± 3.3	6.8 ± 1.1	9.4 ± 2.2	8.3 ± 1.8	11.4 ± 2.2
EMT	6	FrA	contra	2b	4.1 ± 1.5	0.4 ± 1.9	-18.8 ± 1.3	-28.5 ± 2.9	13.44	1.00	1.00	0.0069	4.30e-06	-1.3 ± 3.6	3.4 ± 1.2	22.3 ± 4.3	28.8 ± 6.7	2.8 ± 1.5	3.8 ± 1.9	3.4 ± 1.3	0.3 ± 2.9
EMT	7	PFl	contra	1b	-1.6 ± 1.1	2.2 ± 1.4	6.2 ± 1.6	19.1 ± 2.1	19.77	1.00	1.00	0.19	1.80e-09	-0.6 ± 2.1	-3.8 ± 1.3	-11.5 ± 1.8	-21.5 ± 2.2	-2.2 ± 1.1	-1.6 ± 1.4	-5.3 ± 1.6	-2.5 ± 2.1
EMT	8	PFl	ipsi	1b	-1.8 ± 0.9	-0.9 ± 1.6	9 ± 1.9	15.3 ± 2.9	13.83	1.00	1.00	0.0098	4.01e-07	-2.4 ± 2.2	-5.4 ± 1.5	-15 ± 2	-21.1 ± 2.3	-4.2 ± 0.9	-6.4 ± 1.6	-6 ± 1.9	-5.8 ± 2.9
EMT	9	S1J	contra	2b	7 ± 1.2	0.3 ± 1.1	-14 ± 0.9	-9.2 ± 0.5	16.29	0.63	1.00	1.81e-04	0.0084	-4.1 ± 1.6	0.3 ± 1	12.1 ± 4.1	6.8 ± 1.3	2.9 ± 1.2	0.7 ± 1.1	-1.9 ± 0.9	-2.4 ± 0.5
EMT	10	VS	contra	1b	4.8 ± 1.7	10.9 ± 1.7	20.5 ± 2.9	25.9 ± 2.6	20.58	1.00	0.029	9.84e-08	4.43e-12	1.8 ± 2.7	-1.4 ± 1.6	-2.5 ± 2	-6.8 ± 2.8	6.6 ± 1.7	9.5 ± 1.7	18 ± 2.9	19 ± 2.6
Stroke	1	A	ipsi	2c	9.4 ± 1.1	6.1 ± 1.3	0.3 ± 1.1	-7.1 ± 1.8	18.62	2.96e-04	0.26	1.00	0.0085	-7.3 ± 2.8	-4 ± 1.7	-1.1 ± 2.4	1.4 ± 2.6	2.1 ± 1.1	2.1 ± 1.3	-0.8 ± 1.1	-5.7 ± 1.8
Stroke	2	AI	ipsi	2a	9.9 ± 1.2	4.2 ± 1.1	-3 ± 1	-4.6 ± 1.2	14.08	2.96e-04	0.71	0.73	0.15	-9.2 ± 2.4	-6.3 ± 1.7	-2.9 ± 1.4	-3.4 ± 1.8	0.7 ± 1.2	-2.1 ± 1.1	-5.9 ± 1	-8.1 ± 1.2
Stroke	3	Au1	ipsi	2c	7.4 ± 1.6	-4.3 ± 1.7	-8 ± 1.2	-8.5 ± 1.5	15.05	0.019	0.71	0.020	0.0039	5.5 ± 3.6	6.8 ± 1.7	6.3 ± 2.5	6.2 ± 2.5	12.9 ± 1.6	2.5 ± 1.7	-1.7 ± 1.2	-2.4 ± 1.5
Stroke	4	AuV	ipsi	2a	9.4 ± 1.6	-0.4 ± 1.1	-4.4 ± 1.2	-6.7 ± 1.2	15.24	0.0062	1.00	0.52	0.078	0.2 ± 1.8	1.1 ± 1.1	4.6 ± 1.4	8 ± 1.4	9.6 ± 1.6	0.7 ± 1.1	0.2 ± 1.2	1.2 ± 1.2
Stroke	5	CPu	ipsi	2c	14 ± 2.8	-9.1 ± 1.3	-10.1 ± 1.4	-15.7 ± 1.2	22.43	0.0060	0.26	0.035	6.74e-05	-4.4 ± 1.7	-4.4 ± 1.8	-8 ± 1.7	-4.2 ± 3.5	9.6 ± 2.8	-13.5 ± 1.3	-18.1 ± 1.4	-19.9 ± 1.2
Stroke	6	CPu	contra	2c	8.6 ± 1.2	-2.6 ± 1.2	-4.3 ± 1	-6.7 ± 0.9	20.58	2.96e-04	1.00	0.21	0.0049	-7.2 ± 2.4	-4.7 ± 1.5	-3 ± 1.6	-2.1 ± 1.9	1.4 ± 1.2	-7.4 ± 1.2	-7.2 ± 1	-8.9 ± 0.9
Stroke	7	CPu	ipsi	2c	12.5 ± 2.5	-0.4 ± 2	-9.4 ± 1.9	-16.7 ± 1.8	17.38	0.0060	1.00	0.065	6.04e-05	-7 ± 4.5	-6.1 ± 1.6	-1.1 ± 2.4	3.7 ± 2.6	5.5 ± 2.5	-6.5 ± 2	-10.5 ± 1.9	-13 ± 1.8
Stroke	8	CPu	ipsi	2b	-0.9 ± 1.9	-7.3 ± 1.3	-16.3 ± 1.6	-18.7 ± 2.2	15.87	1.00	0.26	6.92e-08	3.87e-10	1.4 ± 2.1	1.4 ± 1.4	-3.2 ± 0.6	-1.9 ± 1.3	0.6 ± 1.9	-5.9 ± 1.3	-19.5 ± 1.6	-20.5 ± 2.2
Stroke	9	DS	ipsi	2b	6.1 ± 1.7	-7.2 ± 1.8	-13.1 ± 2.2	-18 ± 2.2	16.87	0.22	0.45	0.0029	3.31e-06	-5.4 ± 5.2	-4.5 ± 2.1	2.4 ± 2.4	1.7 ± 3.3	0.7 ± 1.7	-11.7 ± 1.8	-10.7 ± 2.2	-16.3 ± 2.2
Stroke	10	Ect	ipsi	2b	6.6 ± 1.9	-3.2 ± 1.4	-11.2 ± 1.7	-12.1 ± 1.5	19.00	0.10	1.00	9.59e-04	7.28e-05	4 ± 2.8	5 ± 1.8	6 ± 1.3	7.1 ± 1.4	10.7 ± 1.9	1.8 ± 1.4	-5.1 ± 1.7	-5 ± 1.5
Stroke	11	Ect	ipsi	2a	12 ± 2.1	-3.6 ± 1.3	-0.8 ± 1.4	-7.4 ± 1.7	15.86	0.0029	1.00	1.00	0.079	1.1 ± 1.8	6.5 ± 1	2.6 ± 0.9	8.1 ± 0.9	13.1 ± 2.1	2.9 ± 1.3	1.8 ± 1.4	0.7 ± 1.7
Stroke	12	Ent	ipsi	2c	11 ± 1.6	-0.7 ± 1.4	-5.8 ± 1.2	-8.1 ± 1.3	14.85	2.96e-04	1.00	0.14	0.0060	-8.7 ± 1.3	-3.4 ± 2.2	-4 ± 2.1	-4.1 ± 3.1	2.3 ± 1.6	-4.1 ± 1.4	-9.7 ± 1.2	-12.2 ± 1.3
Stroke	13	LAcbSh	ipsi	2c	10.5 ± 2.4	-1.3 ± 1	-9 ± 1.8	-16.3 ± 1.7	16.49	0.018	1.00	0.059	2.55e-05	-8.2 ± 2.7	0 ± 1.3	-0.6 ± 1.3	1.9 ± 2.9	2.3 ± 2.4	-1.3 ± 1	-9.6 ± 1.8	-14.4 ± 1.7
Stroke	14	V2L	ipsi	1c	-13.8 ± 1.4	3.4 ± 3.4	4.2 ± 2.4	20.8 ± 3.9	17.74	0.019	1.00	1.00	6.74e-05	17.1 ± 6.5	6.9 ± 3.6	8.9 ± 1.9	1.8 ± 1.7	3.2 ± 1.4	10.3 ± 3.4	13.1 ± 2.4	22.5 ± 3.9
Stroke	15	VPM	ipsi	2c	23.6 ± 5.1	-11.3 ± 2.5	-18.4 ± 1.7	-20.5 ± 2.3	17.13	0.0062	0.71	0.050	0.0075	1.8 ± 4.7	5.4 ± 6.9	12 ± 5.5	11.9 ± 6.5	25.4 ± 5.1	-5.9 ± 2.5	-6.4 ± 1.7	-8.6 ± 2.3
Stroke+EMT	1	A	ipsi	2c	9.1 ± 1	2.2 ± 0.9	-3.7 ± 1.1	-7.9 ± 0.8	18.97	8.78e-04	1.00	0.74	0.0053	-5.4 ± 2.3	-3.6 ± 1.7	-1 ± 2.3	0.9 ± 2.6	3.7 ± 1	-1.4 ± 0.9	-4.6 ± 1.1	-7 ± 0.8
Stroke+EMT	2	A	contra	2a	13.6 ± 2.5	1.1 ± 0.7	-3.9 ± 1.4	-2.4 ± 1.1	17.40	5.50e-05	1.00	0.75	0.92	-2.9 ± 2.5	-1.9 ± 1.3	-0.9 ± 1.4	-3.3 ± 1.7	10.7 ± 2.5	-0.8 ± 0.7	-4.7 ± 1.4	-5.7 ± 1.1
Stroke+EMT	3	AI	contra	2a	10.7 ± 1.5	0.7 ± 0.6	-0.2 ± 1.2	-2 ± 1.5	14.82	3.17e-07	1.00	1.00	0.84	-5 ± 1.7	1.4 ± 1.9	2.3 ± 1.3	3 ± 0.9	5.6 ± 1.5	2.1 ± 0.6	2.1 ± 1.2	1 ± 1.5
Stroke+EMT	4	AI	ipsi	2c	14.1 ± 4.4	-7.2 ± 2.4	-8.2 ± 2.1	-13.9 ± 2	14.53	0.016	1.00	0.39	0.0022	-5.6 ± 2.4	-3.4 ± 2.6	0.3 ± 1.5	-2.3 ± 1.9	8.5 ± 4.4	-10.6 ± 2.4	-7.9 ± 2.1	-16.2 ± 2
Stroke+EMT	5	AOL	ipsi	1a	-13.2 ± 1.3	7.1 ± 1.2	4 ± 2.5	3.6 ± 2.3	20.21	9.13e-06	0.38	0.75	0.70	8.3 ± 2.2	3.7 ± 1.6	5.4 ± 2.9	3.4 ± 1.9	-4.9 ± 1.3	10.8 ± 1.2	9.4 ± 2.5	7 ± 2.3
Stroke+EMT	6	AOL	contra	1c	-6.5 ± 0.9	4.3 ± 0.9	3.8 ± 1	8.9 ± 0.8	16.72	0.017	0.80	0.48	8.76e-05	5 ± 2.5	-0.2 ± 0.9	-0.8 ± 1.5	-4.3 ± 1.5	-1.6 ± 0.9	4.1 ± 0.9	3 ± 1	4.6 ± 0.8
Stroke+EMT	7	APir	ipsi	2b	3.4 ± 2.6	-7.2 ± 1.5	-15.4 ± 1.8	-27.3 ± 2.3	51.02	0.89	0.63	6.45e-05	8.48e-12	8 ± 2.9	8.1 ± 2.5	15 ± 2.5	25.6 ± 1.6	11.5 ± 2.6	0.9 ± 1.5	-0.4 ± 1.8	-1.7 ± 2.3
Stroke+EMT	8	APir	contra	2b	3.9 ± 1.7	-0.4 ± 1.2	-5.4 ± 1.3	-14.7 ± 1.6	29.33	0.48	1.00	0.26	9.83e-08	1.9 ± 1.2	4.2 ± 0.9	9.6 ± 2.4	17.5 ± 2.8	5.8 ± 1.7	3.8 ± 1.2	4.2 ± 1.3	2.8 ± 1.6
Stroke+EMT	9	APTD	contra	1b	-2.8 ± 1.8	-2.6 ± 1.2	14.9 ± 2.4	26.5 ± 5.5	17.54	1.00	1.00	0.012	1.12e-06	-4.3 ± 4.1	1.6 ± 2.9	-6.3 ± 3.2	-6.4 ± 2.3	-7.1 ± 1.8	-1 ± 1.2	8.6 ± 2.4	20.1 ± 5.5
Stroke+EMT	10	APTD	ipsi	1b	-8.4 ± 2.3	4.1 ± 2.7	17.8 ± 3.2	19.7 ± 4.1	15.16	0.21	1.00	0.0013	1.16e-04	-5.8 ± 2.9	-0.5 ± 2.7	-6.7 ± 2.9	-6.2 ± 3.1	-14.2 ± 2.3	3.6 ± 2.7	11.1 ± 3.2	13.5 ± 4.1
Stroke+EMT	11	AuV	ipsi	2b	1.1 ± 1.7	-7.7 ± 1.9	-15.2 ± 1.8	-20.5 ± 1.4	25.62	1.00	0.18	3.24e-06	8.07e-11	-0.6 ± 2	0.3 ± 1	4.3 ± 1.3	7.1 ± 1.3	0.6 ± 1.7	-7.3 ± 1.9	-11 ± 1.8	-13.4 ± 1.4
Stroke+EMT	12	AuV	contra	2b	7.9 ± 3.1	-7.7 ± 1.3	-7.9 ± 1	-12.5 ± 1.5	17.44	0.085	0.36	0.099	1.78e-04	1.2 ± 1.7	5.2 ± 1.6	5.8 ± 2.2	8.9 ± 1.7	9.2 ± 3.1	-2.5 ± 1.3	-2.1 ± 1	-3.6 ± 1.5
Stroke+EMT	13	AVVL	contra	1b	-8.2 ± 2.1	6.4 ± 4.8	26.3 ± 6.8	30.9 ± 6.2	17.72	0.72	1.00	0.0018	1.35e-05	-8.7 ± 5.4	-3.1 ± 5.2	-8 ± 3.5	-14.8 ± 2.8	-16.9 ± 2.1	3.3 ± 4.8	18.3 ± 6.8	16.2 ± 6.2
Stroke+EMT	14	Cb2	contra	2c	23.5 ± 7.7	-7.9 ± 3.7	-5.3 ± 3.1	-24.1 ± 3	18.02	0.016	1.00	0.99	5.94e-04	2.3 ± 5.4	3.5 ± 3.9	0.6 ± 4.3	9.6 ± 3.9	25.9 ± 7.7	-4.4 ± 3.7	-4.6 ± 3.1	-14.5 ± 3
Stroke+EMT	15	Cb2/3	ipsi	2a	21.8 ± 4.3	-4.6 ± 2.5	-1.9 ± 2.6	-9.4 ± 2.8	18.19	3.81e-04	1.00	1.00	0.18	1.1 ± 4.6	2.1 ± 3.1	-3.4 ± 3.7	-2 ± 3.8	22.9 ± 4.3	-2.5 ± 2.5	-5.3 ± 2.6	-11.4 ± 2.8
Stroke+EMT	16	Cb2b	contra	2c	11 ± 2.2	-6.8 ± 1.5	-10.5 ± 1.2	-12.7 ± 1.6	19.40	0.019	1.00	0.068	0.0030	-0.9 ± 3.8	4.3 ± 3.2	5.7 ± 4.1	3.9 ± 4.1	10.1 ± 2.2	-2.4 ± 1.5	-4.8 ± 1.2	-8.8 ± 1.6
Stroke+EMT	17	Cb5	ipsi	2c	21.4 ± 2.4	1.1 ± 3.2	-8 ± 2.2	-17 ± 2.1	28.73	3.41e-04	1.00	0.67	0.0040	-2.2 ± 2.8	2.1 ± 3	7 ± 2.4	10.8 ± 5.1	19.1 ± 2.4	3.2 ± 3.2	-1 ± 2.2	-6.2 ± 2.1
Stroke+EMT	18	CPu	ipsi	2c	43.9 ± 5.7	4.7 ± 2	-6.1 ± 3.5	-10.3 ± 2.9	42.36	9.21e-13	1.00	0.64	0.035	-3.3 ± 3.2	-2.1 ± 1.7	2 ± 0.9	3.2 ± 2	40.5 ± 5.7	2.6 ± 2	-4.1 ± 3.5	-7.1 ± 2.9
Stroke+EMT	19	CPu	ipsi	2c	40.5 ± 5.8	7.3 ± 4	-5 ± 2.5	-11.3 ± 2	72.93	9.21e-13	1.00	0.75	0.0078	-5.3 ± 2.4	-4.2 ± 1.1	-2.1 ± 1.4	-1.4 ± 1.7	35.1 ± 5.8	3.1 ± 4	-7 ± 2.5	-12.7 ± 2
Stroke+EMT	20	CPu	ipsi	2a	88.5 ± 13.2	-0.9 ± 4.6	-6 ± 5.1	-5 ± 2.6	60.45	4.95e-13	1.00	0.81	0.88	-8.3 ± 3.6	-8.2 ± 1.1	-2.7 ± 2.4	-4.2 ± 2.5	80.2 ± 13.2	-9.1 ± 4.6	-8.7 ± 5.1	-9.2 ± 2.6
Stroke+EMT	21	CPu	contra	2a	23.6 ± 3.3	6.7 ± 1.4	-2.2 ± 2	-2.3 ± 1.8	29.09	4.57e-11	0.63	1.00	0.93	-6 ± 2.9	-4.2 ± 2.1	1.2 ± 3.3	-0.5 ± 1.6	17.6 ± 3.3	2.5 ± 1.4	-1.1 ± 2	-2.7 ± 1.8
Stroke+EMT	22	CPu	ipsi	2c	24.3 ± 4.1	-1.6 ± 2.5	-8.4 ± 1.7	-13.7 ± 2.3	28.06	7.97e-07	1.00	0.26	0.0013	-2.4 ± 2	2.2 ± 2.8	-0.5 ± 2.1	1.3 ± 1.2	21.9 ± 4.1	0.7 ± 2.5	-9 ± 1.7	-12.4 ± 2.3
Stroke+EMT	23	CPu	contra	2a	24.2 ± 6.2	1.2 ± 3	-5.1 ± 1.6	-3.7 ± 1.5	19.50	2.29e-08	1.00	0.75	0.85	-5.2 ± 0.6	-0.5 ± 0.9	0.9 ± 3.6	-0.2 ± 3.1	19 ± 6.2	0.7 ± 3	-4.2 ± 1.6	-3.9 ± 1.5
Stroke+EMT	24	CPu	contra	1a	-22.2 ± 2.9	-4.4 ± 1.6	7.9 ± 3.1	7.6 ± 3.6	16.53	6.94e-05	1.00	0.78	0.71	-2.2 ± 4.5	2.4 ± 3.8	-1.1 ± 3.1	3.9 ± 4.4	-24.4 ± 2.9	-2 ± 1.6	6.8 ± 3.1	11.5 ± 3.6
Stroke+EMT	25	CPu	contra	1c	-15 ± 2.9	0.1 ± 1.5	10.7 ± 2.8	12.8 ± 3.4	16.13	0.0035	1.00	0.21	0.030	-0.3 ± 2.7	-4.9 ± 1.4	-8 ± 0.9	-9.2 ± 1.7	-15.3 ± 2.9	-4.8 ± 1.5	2.7 ± 2.8	3.6 ± 3.4
Stroke+EMT	26	CPu	contra	1c	-9.6 ± 1.3	2 ± 1.9	7.3 ± 2.3	10.9 ± 2.1	13.81	0.011	1.00	0.19	0.0019	3.7 ± 3	-0.3 ± 1.6	-2.1 ± 1.7	-4.3 ± 1.4	-5.9 ± 1.3	1.7 ± 1.9	5.2 ± 2.3	6.6 ± 2.1
Stroke+EMT	27	Crus	ipsi	2b	6.5 ± 2.7	0.9 ± 1.1	-4.7 ± 1.7	-20.4 ± 2.2	23.03	0.26	1.00	0.75	7.32e-08	6.7 ± 3.9	-0.6 ± 2.3	5.5 ± 2.7	21.6 ± 2.7	13.3 ± 2.7	0.2 ± 1.1	0.8 ± 1.7	1.1 ± 2.2
Stroke+EMT	28	Crus	ipsi	2b	0.1 ± 1.4	1.6 ± 1.3	4.5 ± 2	-19.4 ± 1.8	19.28	1.00	1.00	0.72	1.81e-07	-5.2 ± 1	-8.1 ± 1.5	-12.9 ± 2.2	8.4 ± 5.2	-5.1 ± 1.3	-6.5 ± 1.3	-8.4 ± 2	-11 ± 1.8
Stroke+EMT	29	Crus	contra	2b	0.1 ± 2.1	-1.6 ± 1.4	-0.1 ± 2.3	-24.3 ± 1.5	18.89	1.00	1.00	1.00	3.85e-09	-6.5 ± 1.2	-7.8 ± 0.7	-9.4 ± 2.6	10.1 ± 5.6	-6.4 ± 2.1	-9.4 ± 1.4	-9.6 ± 2.3	-14.2 ± 1.5

Group	Event	Area	Hemisphere	Profile	Effect size Week				F value	q-value Week				Volume change Control Week				Volume change Group Week			
					1	2	4	8		1	2	4	8	1	2	4	8	1	2	4	8
Stroke+EMT	30	DCDp	contra	2b	10.8 ± 2.9	-4.7 ± 2.3	-3.9 ± 1.7	-25.8 ± 1.7	28.92	0.062	1.00	0.94	1.12e-08	-4.9 ± 1.4	-2.4 ± 1.8	-2.7 ± 2.4	12.4 ± 4	5.9 ± 2.9	-7 ± 2.3	-6.5 ± 1.7	-13.4 ± 1.7
Stroke+EMT	31	DCDp	ipsi	2b	9.1 ± 2.5	-5.2 ± 1.6	-7.1 ± 1.4	-25.3 ± 1.5	25.90	0.11	1.00	0.37	8.81e-10	-1.1 ± 1.8	-1.4 ± 1.1	-1.1 ± 2.1	11.7 ± 4.7	8 ± 2.5	-6.6 ± 1.6	-8.2 ± 1.4	-13.7 ± 1.5
Stroke+EMT	32	DCIC	contra	2b	8.2 ± 3.9	-0.4 ± 3.4	-6.2 ± 3.3	-25.3 ± 2.6	21.30	0.54	1.00	0.81	4.09e-06	2.8 ± 2.9	5.8 ± 2.6	10.3 ± 2.8	19.1 ± 5.1	10.9 ± 3.9	5.4 ± 3.4	4.1 ± 3.3	-6.2 ± 2.6
Stroke+EMT	33	Ent	ipsi	2b	1 ± 1	-2.8 ± 1.5	-12.5 ± 1.4	-23.8 ± 2	35.20	1.00	1.00	0.0012	1.25e-10	6.9 ± 2.3	8 ± 1.2	18.8 ± 2.4	30.5 ± 2.4	7.9 ± 1	5.1 ± 1.5	6.3 ± 1.4	6.7 ± 2
Stroke+EMT	34	Ent	ipsi	2c	23.1 ± 2.7	2 ± 1.7	-5.9 ± 1.8	-11.4 ± 1.6	29.58	8.61e-08	1.00	0.75	0.014	-7.2 ± 2.7	-3.1 ± 1	4.5 ± 3.5	6.6 ± 2.6	16 ± 2.7	-1 ± 1.7	-1.4 ± 1.8	-4.8 ± 1.6
Stroke+EMT	35	Ent	contra	2b	1.8 ± 2.4	-0.5 ± 2.8	-8.6 ± 2	-20.7 ± 2	23.18	1.00	1.00	0.37	7.24e-06	7 ± 3.2	8.3 ± 3	17 ± 4.2	26 ± 4.1	8.8 ± 2.4	7.8 ± 2.8	8.4 ± 2	5.3 ± 2
Stroke+EMT	36	Ent	ipsi	1b	-3.5 ± 1.1	3.8 ± 1	1.8 ± 0.8	10.7 ± 1.4	20.12	0.52	1.00	0.98	2.83e-06	1.3 ± 1.1	-3.3 ± 1.2	-3.7 ± 1.4	-10 ± 1.5	-2.1 ± 1.1	0.5 ± 1	-1.9 ± 0.8	0.6 ± 1.4
Stroke+EMT	37	Ent	contra	2b	3 ± 1.6	-0.2 ± 1.4	-6.9 ± 1.3	-11.7 ± 2.2	14.61	0.77	1.00	0.059	9.90e-06	0.6 ± 1.3	1 ± 1.5	5.7 ± 2.8	9.2 ± 2.2	3.6 ± 1.6	0.8 ± 1.4	-1.2 ± 1.3	-2.4 ± 2.2
Stroke+EMT	38	FrA	ipsi	2b	6.1 ± 3.1	-4.2 ± 3	-20.7 ± 2.2	-24.4 ± 2.8	20.10	0.75	1.00	2.47e-04	2.19e-06	0.3 ± 2.2	3.4 ± 2.2	14.7 ± 4.3	15.9 ± 4.8	6.4 ± 3.1	-0.8 ± 3	-6 ± 2.2	-8.5 ± 2.8
Stroke+EMT	39	FrA	contra	2b	2.2 ± 1.9	-0.1 ± 2	-20 ± 1.6	-29.3 ± 3.1	14.31	1.00	1.00	0.0042	2.38e-06	-1.3 ± 3.4	3.7 ± 1.1	21.2 ± 3.8	28.2 ± 6.4	0.9 ± 1.9	3.6 ± 2	1.2 ± 1.6	-1.1 ± 3.1
Stroke+EMT	40	GrO	ipsi	1b	-9.3 ± 2	4.1 ± 3.6	6 ± 3.5	16.3 ± 3.9	21.44	0.086	1.00	0.72	1.94e-04	2.6 ± 2.4	-0.7 ± 3	-3.9 ± 3.4	-8.5 ± 1.9	-6.7 ± 2	3.4 ± 3.6	2 ± 3.5	7.8 ± 3.9
Stroke+EMT	41	GrO	contra	1b	-3.6 ± 1.5	1.4 ± 2.8	6.3 ± 2.3	14.6 ± 2.7	17.74	0.78	1.00	0.32	1.67e-05	-1.3 ± 1.3	-3 ± 2.1	-8.7 ± 2.2	-10.9 ± 1.4	-5 ± 1.5	-1.5 ± 2.8	-2.3 ± 2.3	3.7 ± 2.7
Stroke+EMT	42	HC	ipsi	2a	92 ± 14.1	26.3 ± 5.9	-5.8 ± 3.3	0 ± 3.6	47.71	4.95e-13	0.0042	0.86	1.00	-4 ± 2.8	-2.5 ± 1.9	-0.2 ± 2	-0.6 ± 1.7	88.1 ± 14.1	23.9 ± 5.9	-5.9 ± 3.3	-0.7 ± 3.6
Stroke+EMT	43	HC	contra	2b	8.5 ± 5.9	-8.4 ± 1.8	-6.5 ± 2.9	-26 ± 1.7	15.84	0.73	1.00	0.87	6.29e-05	12.4 ± 4.3	13.3 ± 5.8	12.8 ± 4.7	25.3 ± 5.2	21 ± 5.9	4.8 ± 1.8	6.2 ± 2.9	-0.7 ± 1.7
Stroke+EMT	44	InG	contra	2a	22.6 ± 5.1	-1.4 ± 1.7	-3.9 ± 1.7	-2.3 ± 2.1	24.99	8.57e-09	1.00	0.81	0.93	-1.2 ± 1.5	0.8 ± 0.9	2.1 ± 1.7	0.5 ± 1.5	21.4 ± 5.1	-0.5 ± 1.7	-1.7 ± 1.7	-1.8 ± 2.1
Stroke+EMT	45	LPtA	ipsi	1c	-10.9 ± 1.5	1.6 ± 2.5	17.3 ± 4.1	20.4 ± 4.9	25.80	0.011	1.00	4.74e-05	4.68e-07	7.1 ± 2	0.9 ± 1.2	-4.4 ± 1.2	-4.4 ± 1.3	-3.7 ± 1.5	2.4 ± 2.5	12.9 ± 4.1	16.1 ± 4.9
Stroke+EMT	46	LS	ipsi	1c	-10.5 ± 3.1	7.6 ± 2.6	21.2 ± 3.3	27.1 ± 5.2	20.59	0.049	1.00	3.79e-04	1.26e-06	-1.4 ± 1.9	-3.2 ± 2.1	2.4 ± 2.2	-0.1 ± 1.1	-11.9 ± 3.1	4.3 ± 2.6	23.5 ± 3.3	27 ± 5.2
Stroke+EMT	47	LS	contra	2a	24.4 ± 5.2	2.7 ± 1.9	-7.2 ± 2.4	-10.4 ± 2	17.48	1.05e-05	1.00	0.65	0.10	-0.8 ± 2.9	1.2 ± 2.6	4.7 ± 2.7	8.4 ± 2.7	23.6 ± 5.2	3.9 ± 1.9	-2.5 ± 2.4	-2 ± 2
Stroke+EMT	48	LVPO	ipsi	2c	19 ± 4.2	-9.5 ± 3.3	-14.2 ± 2.1	-12.5 ± 3	21.06	0.0043	0.91	0.041	0.044	-6.2 ± 4.5	-1.5 ± 3.7	-3.3 ± 3.7	-2.1 ± 3.2	12.8 ± 4.2	-11 ± 3.3	-17.5 ± 2.1	-14.6 ± 3
Stroke+EMT	49	LVPO	contra	2a	19.9 ± 4.7	-2.6 ± 3.5	-8.5 ± 2.8	-13.4 ± 3.8	15.19	0.018	1.00	0.74	0.11	-9.7 ± 4	-5.6 ± 3	-6.6 ± 2.2	-2.7 ± 4.5	10.1 ± 4.7	-8.2 ± 3.5	-15.1 ± 2.8	-16 ± 3.8
Stroke+EMT	50	PFl	ipsi	1b	-1.4 ± 1.1	5.1 ± 1	13.5 ± 1.2	16.9 ± 1.5	21.21	1.00	0.97	2.96e-05	4.07e-09	-5.5 ± 1.9	-9.1 ± 1.9	-16.4 ± 1.7	-22.8 ± 1.4	-6.9 ± 1.1	-4 ± 1	-2.9 ± 1.2	-5.9 ± 1.5
Stroke+EMT	51	PFl	contra	1b	-5.3 ± 1.1	-1.6 ± 1.7	3.6 ± 1.2	13.7 ± 1.4	16.75	0.61	1.00	0.83	1.00e-04	-0.8 ± 2.4	-2.7 ± 1.7	-8.3 ± 3.8	-13.9 ± 4.1	-6.1 ± 1.1	-4.3 ± 1.7	-4.8 ± 1.2	-0.2 ± 1.4
Stroke+EMT	52	PFl	contra	1b	-5.9 ± 1	-0.1 ± 1.6	5.6 ± 1.8	15 ± 2.3	16.64	0.34	1.00	0.48	2.38e-06	2.7 ± 1.9	-0.8 ± 0.6	-6.8 ± 1.8	-14.7 ± 2.2	-3.2 ± 1	-0.9 ± 1.6	-1.2 ± 1.8	0.4 ± 2.3
Stroke+EMT	53	PFl	ipsi	1b	-1.5 ± 1.7	-0.5 ± 1.8	10.2 ± 2	16.1 ± 3.4	16.52	1.00	1.00	0.0042	1.66e-07	-1.5 ± 2.2	-5.5 ± 1.2	-13.6 ± 1.6	-18.7 ± 1.8	-2.9 ± 1.7	-6 ± 1.8	-3.5 ± 2	-2.5 ± 3.4
Stroke+EMT	54	PFl	contra	2b	5.4 ± 2.3	-6.7 ± 1.7	-11.4 ± 2.2	-22.7 ± 1.7	15.32	0.71	1.00	0.048	9.63e-07	3.4 ± 2.7	3.2 ± 3.4	9 ± 3.8	20.6 ± 4.4	8.8 ± 2.3	-3.5 ± 1.7	-2.5 ± 2.2	-2 ± 1.7
Stroke+EMT	55	Pir	contra	2a	24.7 ± 4.4	3.4 ± 2.9	0.7 ± 1.8	-2.9 ± 1.1	24.75	8.58e-12	1.00	1.00	0.88	-11.6 ± 1.9	-3.3 ± 1.7	0.5 ± 1.7	-0.9 ± 1.4	13.2 ± 4.4	0 ± 2.9	1.1 ± 1.8	-3.8 ± 1.1
Stroke+EMT	56	Pir	ipsi	2a	19.2 ± 3.4	-2.9 ± 1.7	-2.8 ± 2.4	-5.9 ± 1.4	24.17	5.84e-08	1.00	0.97	0.25	-9.4 ± 1.9	-0.9 ± 1.4	4.5 ± 1.8	1.9 ± 1.4	9.8 ± 3.4	-3.7 ± 1.7	1.7 ± 2.4	-4 ± 1.4
Stroke+EMT	57	Pir	contra	1b	-7 ± 1.4	6.4 ± 1.9	4.1 ± 1.8	13.4 ± 3	15.44	0.14	0.76	0.75	8.32e-05	7 ± 2.4	1.4 ± 1.6	2.2 ± 3	-2.9 ± 1.5	0 ± 1.4	7.7 ± 1.9	6.3 ± 1.8	10.6 ± 3
Stroke+EMT	58	Pir	contra	1c	-12.1 ± 1.1	1.5 ± 1.2	2.6 ± 1	12.7 ± 2.8	13.88	0.015	1.00	0.96	2.87e-04	12.7 ± 4.6	5.7 ± 1.3	5.5 ± 3.3	0.9 ± 2	0.6 ± 1.1	7.3 ± 1.2	8.1 ± 1	13.6 ± 2.8
Stroke+EMT	59	PLH	contra	1c	-9.3 ± 2.6	10.4 ± 1.8	14.4 ± 2.4	12 ± 2.2	24.05	0.016	0.036	9.93e-05	8.68e-04	5.4 ± 4.6	-0.7 ± 1.1	-0.9 ± 1	2.9 ± 1.9	-3.9 ± 2.6	9.6 ± 1.8	13.5 ± 2.4	14.9 ± 2.2
Stroke+EMT	60	Pn	ipsi	2a	36.6 ± 6.3	6.7 ± 4.8	-2 ± 4.1	-10 ± 2.4	23.26	4.98e-07	1.00	1.00	0.33	-10.7 ± 4.7	-1.7 ± 2.4	-4.5 ± 4.3	-4.6 ± 3.4	25.9 ± 6.3	5 ± 4.8	-6.5 ± 4.1	-14.5 ± 2.4
Stroke+EMT	61	PRh	ipsi	2b	4.6 ± 3	-3.3 ± 1.6	-11.7 ± 1.8	-19 ± 1.5	28.60	0.73	1.00	0.012	2.80e-06	5.5 ± 1.9	7.8 ± 1.3	18.7 ± 2.9	26.3 ± 3.8	10.1 ± 3	4.5 ± 1.6	7 ± 1.8	7.4 ± 1.5
Stroke+EMT	62	RS	ipsi	2a	43.4 ± 8.2	0.2 ± 1.9	-5.8 ± 1.6	-7.5 ± 2.8	29.75	2.39e-08	1.00	0.88	0.58	-3.8 ± 2.9	-10.3 ± 3.3	-5.5 ± 1.9	-4.4 ± 2.5	39.6 ± 8.2	-10.1 ± 1.9	-11.2 ± 1.6	-11.9 ± 2.8
Stroke+EMT	63	RS	contra	2c	17.3 ± 5.1	-2.1 ± 1.9	-18.1 ± 2.8	-12.5 ± 3.3	19.24	0.010	1.00	0.0015	0.026	-8 ± 3.1	-12.5 ± 1.4	-0.5 ± 3.1	-4.1 ± 1.7	9.3 ± 5.1	-14.6 ± 1.9	-18.5 ± 2.8	-16.6 ± 3.3
Stroke+EMT	64	Rt	ipsi	1b	11 ± 3.5	5.2 ± 3.2	9.9 ± 6.7	67 ± 16.2	23.44	0.48	1.00	0.81	1.68e-10	2.9 ± 4.9	3.7 ± 2.8	5.9 ± 2.5	5.9 ± 1.9	13.9 ± 3.5	8.9 ± 3.2	15.7 ± 6.7	72.9 ± 16.2
Stroke+EMT	65	S1BF	ipsi	2c	15.8 ± 4.8	-17.5 ± 1.8	-15.4 ± 2.3	-17.2 ± 2.2	47.98	5.32e-05	6.92e-07	3.24e-06	3.05e-08	-8.8 ± 1.4	-3.6 ± 1.8	-6.9 ± 1	-6.2 ± 1	7.1 ± 4.8	-21.1 ± 1.8	-22.3 ± 2.3	-23.5 ± 2.2
Stroke+EMT	66	S1BF	contra	2c	8.5 ± 1.5	0.6 ± 0.7	-2.4 ± 1.2	-6.4 ± 1.4	17.82	2.53e-04	1.00	0.81	0.0031	-5 ± 1.4	-3.5 ± 0.9	-3.5 ± 1.1	-4.4 ± 1.2	3.5 ± 1.5	-3 ± 0.7	-6 ± 1.2	-10.8 ± 1.4
Stroke+EMT	67	S1BF	ipsi	2b	8.9 ± 6.6	-13.3 ± 2.5	-15.9 ± 2.6	-14.4 ± 2.2	15.41	0.31	0.0042	4.72e-05	6.96e-05	-5.6 ± 2.4	-7.1 ± 0.9	-6.6 ± 1.4	-7.8 ± 1.3	3.2 ± 6.6	-20.4 ± 2.5	-22.5 ± 2.6	-22.2 ± 2.2
Stroke+EMT	68	S1FL	ipsi	1c	-17.1 ± 1.2	-3.4 ± 1.6	1.4 ± 2.5	10.5 ± 3.3	20.21	4.02e-07	1.00	1.00	0.0034	7.8 ± 4.8	-0.1 ± 1	-3.4 ± 1.6	-5.2 ± 1.3	-9.3 ± 1.2	-3.5 ± 1.6	-2 ± 2.5	5.3 ± 3.3
Stroke+EMT	69	S1HL	ipsi	1a	-60.2 ± 3.8	9.1 ± 2.3	84.5 ± 8.1	20.3 ± 6.2	86.17	4.95e-13	1.00	3.63e-11	0.080	5 ± 2.8	-6.6 ± 4.2	-6.2 ± 3.5	-11.3 ± 4	-55.2 ± 3.8	2.5 ± 2.3	78.3 ± 8.1	9 ± 6.2
Stroke+EMT	70	S1HL	ipsi	2a	52.1 ± 8.7	-5.2 ± 3.4	-20 ± 6.9	-6.4 ± 7.9	22.03	5.03e-07	1.00	0.012	0.84	-7.2 ± 3.6	0.6 ± 3.1	0 ± 3.5	7 ± 4.9	44.9 ± 8.7	-4.6 ± 3.4	-19.9 ± 6.9	0.7 ± 7.9
Stroke+EMT	71	S1ULp	contra	1c	-5.1 ± 1.6	3.5 ± 2	3.3 ± 1.2	10 ± 2	24.18	0.048	1.00	0.64	8.57e-06	0.6 ± 1	0.1 ± 0.6	0.3 ± 1.3	-0.8 ± 1.2	-4.5 ± 1.6	3.6 ± 2	3.6 ± 1.2	9.1 ± 2
Stroke+EMT	72	S1ULp	ipsi	2c	14.5 ± 5.4	0.1 ± 4.5	-8.9 ± 3.9	-11.9 ± 2.9	18.21	0.016	1.00	0.21	0.015	-7.6 ± 1.3	-3.2 ± 2.3	0 ± 1.5	-0.4 ± 2	6.9 ± 5.4	-3 ± 4.5	-9 ± 3.9	-12.3 ± 2.9
Stroke+EMT	73	S1ULp	contra	1b	-3.5 ± 0.7	4.1 ± 2.1	2.2 ± 0.9	10.7 ± 1.8	16.91	0.40	0.91	0.86	2.38e-06	-2 ± 1	-2.2 ± 0.9	-0.9 ± 2.3	-4.4 ± 0.9	-5.5 ± 0.7	1.9 ± 2.1	1.3 ± 0.9	6.3 ± 1.8
Stroke+EMT	74	S1ULp	ipsi	1c	-11.5 ± 2.4	-1.8 ± 3.1	5.8 ± 4.2	11.3 ± 3.2	13.45	0.0039	1.00	0.75	0.010	8.6 ± 2.1	7.2 ± 1.2	5.2 ± 1.4	4.6 ± 1.2	-3 ± 2.4	5.4 ± 3.1	11 ± 4.2	15.9 ± 3.2
Stroke+EMT	75	S2	ipsi	2b	3.2 ± 3.5	-5.9 ± 2.3	-14.4 ± 1.9	-19.8 ± 1.5	23.27	0.89	0.68	5.12e-06	1.19e-10	5 ± 3	5 ± 1	5.6 ± 1	8.4 ± 1.2	8.2 ± 3.5	-0.8 ± 2.3	-8.8 ± 1.9	-11.4 ± 1.5
Stroke+EMT	76	S2	contra	1c	-9.3 ± 0.8	3.8 ± 1.5	3.1 ± 1	6 ± 0.8	34.48	8.01e-07	0.70	0.58	0.0045	4 ± 1.1	0.6 ± 0.9	0.5 ± 1.1	1.8 ± 1.5	-5.4 ± 0.8	4.4 ± 1.5	3.6 ± 1	7.7 ± 0.8
Stroke+EMT	77	Tu1	contra	2a	19.3 ± 3.5	6.8 ± 1.5	0.4 ± 1.2	-1.4 ± 1.2	19.07	4.26e-09	0.47	1.00	0.99	-3.5 ± 1.8	-1.1 ± 1.4	2.1 ± 2.3	3 ± 1.4	15.9 ± 3.5	5.8 ± 1.5	2.5 ± 1.2	1.5 ± 1.2
Stroke+EMT	78	Tu1	ipsi	1c	-15.3 ± 2.3	7.6 ± 4.3	9.7 ± 3.9	20.6 ± 4.4	16.82	0.049	1.00	0.41	1.92e-04	19.4 ± 10.5	3.9 ± 1.2	5.7 ± 2.7	0.6 ± 1.1	4.1 ± 2.3	11.4 ± 4.3	15.5 ± 3.9	21.1 ± 4.4
Stroke+EMT	79	V1	ipsi	1c	-9.9 ± 1.7	6.4 ± 1.5	9.2 ± 1.6	8.1 ± 1.6	19.13	0.011	0.63	0.012	0.020	7 ± 5.4	-1.9 ± 1.1	-6.1 ± 1.7	-6.3 ± 0.9	-3 ± 1.7	4.5 ± 1.5	3 ± 1.6	1.8 ± 1.6
Stroke+EMT	80	V2L	ipsi	2b	5.5 ± 1.4	-9.5 ± 1.6	-8.2 ± 1.9	-15.5 ± 0.7	17.16	0.30	0.063	0.059	3.04e-06	2 ± 2.5	10.9 ± 1.7	8.9 ± 2.4	16.2 ± 1.8	7.6 ± 1.4	1.5 ± 1.6	0.7 ± 1.9	0.7 ± 0.7
Stroke+EMT	81	Ve	ipsi	1b	-5.8 ± 1.4	-1.4 ± 1.8	8.6 ± 2.2	18.3 ± 2.7	18.08	0.48	1.00	0.16	5.13e-07	-5.3 ± 1.7	-5.8 ± 1.6	-11.6 ± 1.8	-19.8 ± 3.5	-11.1 ± 1.4	-7.1 ± 1.8	-3 ± 2.2	-1.5 ± 2.7
Stroke+EMT	82	VS	contra	1b	-2.5 ± 1.2	10.1 ± 1.3	13.1 ± 1.7	17.4 ± 2.1	21.93	0.97	0.030	1.23e-04	2.73e-08	2.4 ± 2.1	-1.1 ± 1.4	-2 ± 2.1	-6.3 ± 2.8	-0.2 ± 1.3	9 ± 1.3	11.1 ± 1.7	11 ± 2.1

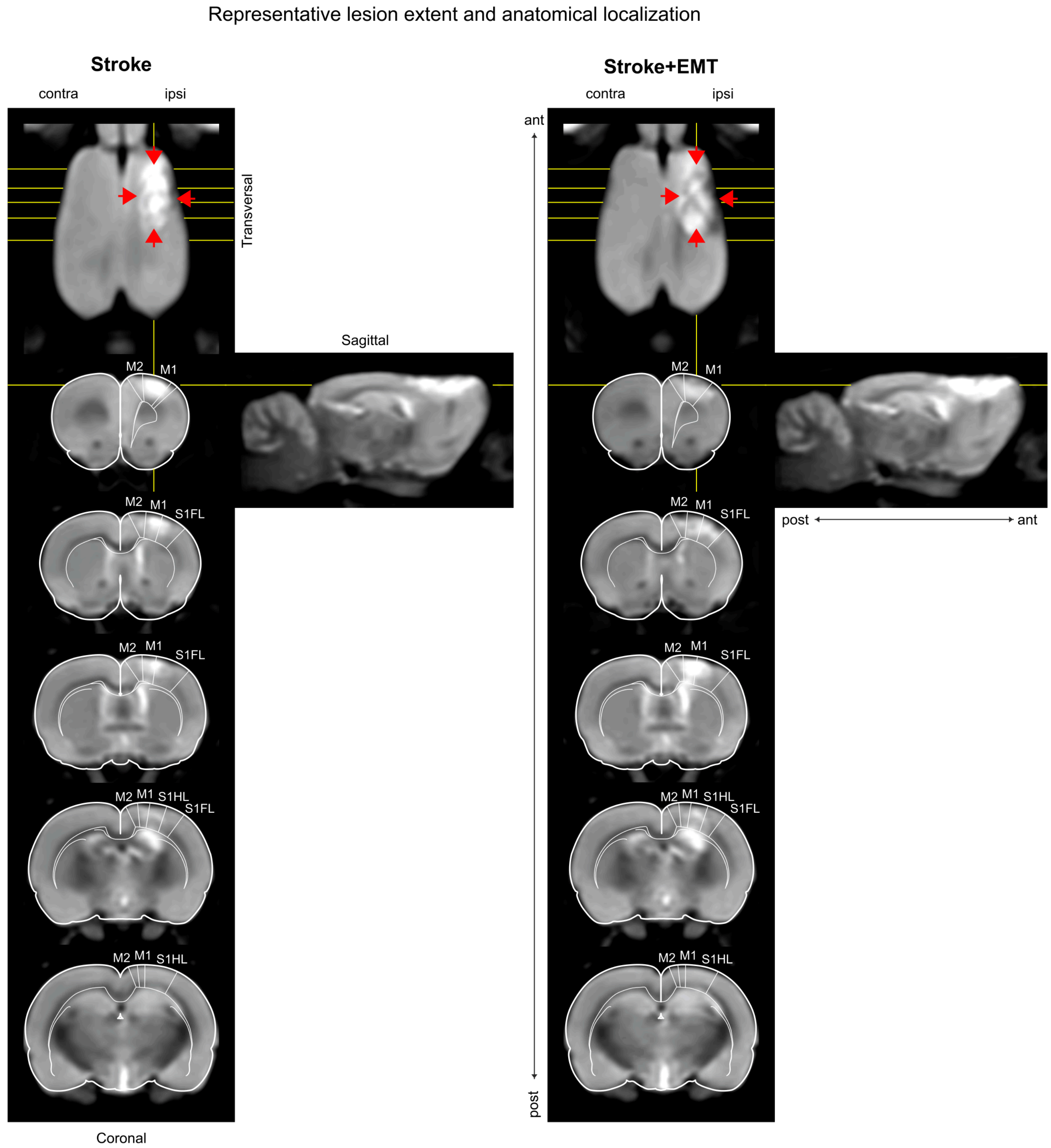

Figure S1. Representative T2-weighted MRI sections illustrating lesion extent and anatomical localization in the Stroke and Stroke+EMT groups. Transversal, sagittal, and selected coronal sections of T2-weighted MR images, averaged across all Stroke or Stroke+EMT rats at Week 8 post stroke, illustrate the position and extent of the lesion (red arrows in transversal views). Abbreviations: M1 indicates primary motor cortex; M2, secondary motor cortex; S1FL, primary somatosensory forelimb area; and S1HL, primary somatosensory hindlimb area.

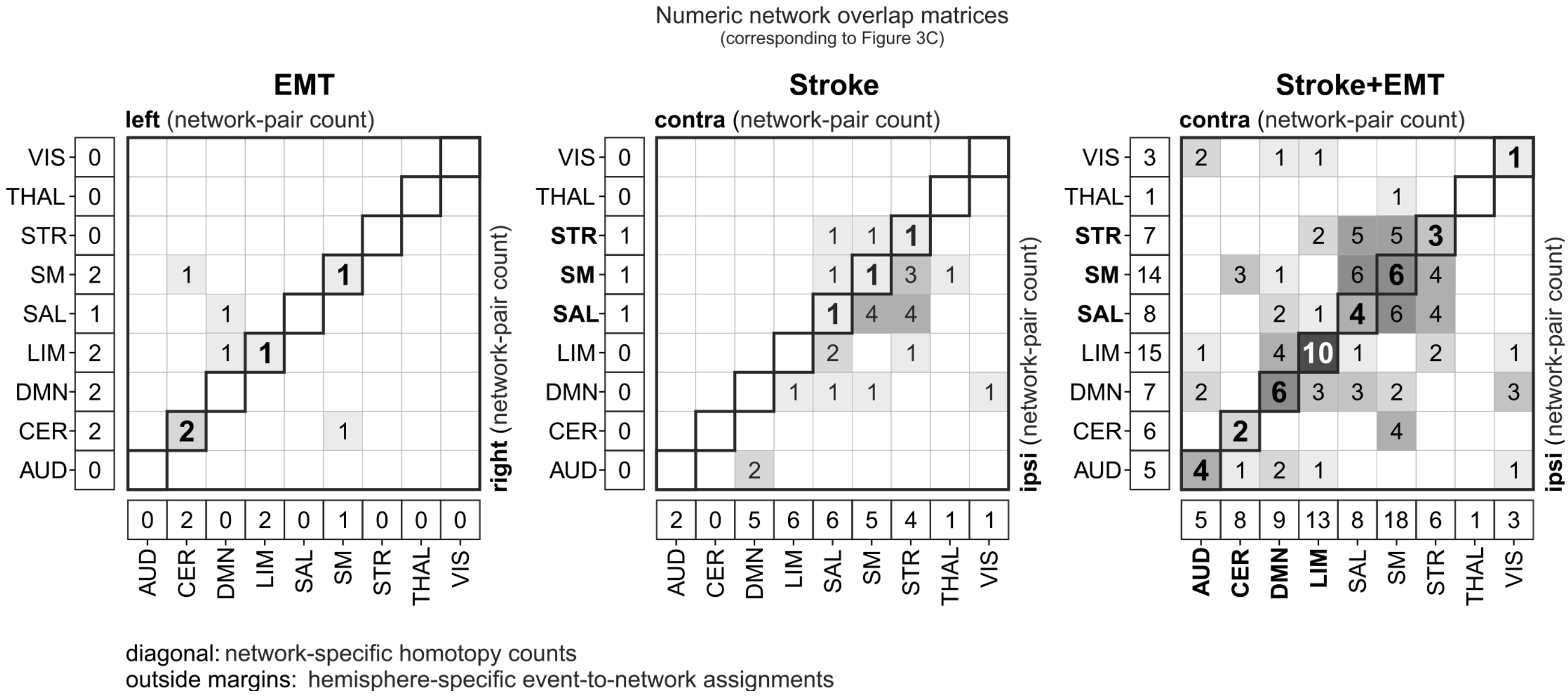

Figure S2. Hemisphere-resolved numeric overlap matrices related to Figure 3C. Cell numbers in upper-left (contralateral) or lower-right (ipsilateral) triangles indicate how often structural events were assigned to individual pairs of networks within the same hemisphere (network-pair counts). Numbers in diagonal cells indicate how often event-bearing homotopic area pairs contributed to the respective network (network-specific homotopy counts). Numbers outside the matrix indicate how often structural events were assigned to the respective network in the corresponding hemisphere (event-to-network assignments). Abbreviations: AUD indicates auditory; CER, cerebellar; DMN, default mode; LIM, limbic; SAL, salience; SM, sensorimotor; STR, striatal; THAL, thalamic; and VIS, visual network category.

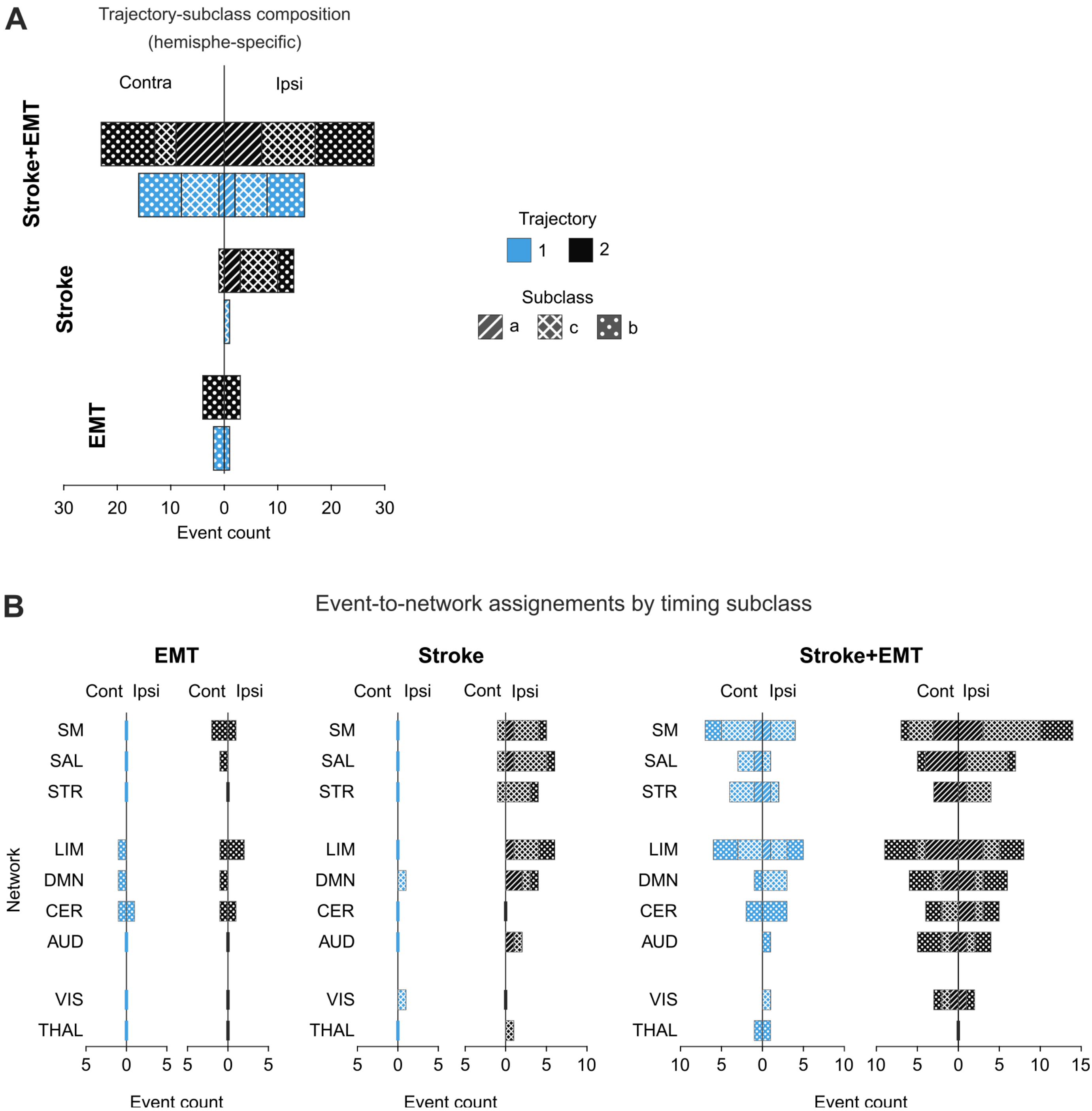

Figure S3. Hemisphere-resolved temporal-profile composition and subclass-specific event-to-network assignments related to Figure 4. (A) Structural events were counted once per event and summarized separately by hemisphere for each experimental group. Stacked bars show the hemisphere-resolved composition across the 6 temporal profiles generated by combining the 2 EWEP-derived main trajectories (T1, T2) with the timing-defined subclasses a, b, and c. This panel provides the hemisphere-resolved denominator underlying the group-level composition shown in Figure 4A2. (B) Event-to-network assignments were summarized separately by hemisphere and displayed by timing-defined subclass across the study-specific network categories for each experimental group. Because area-to-network assignment was non-exclusive, individual structural events could contribute to more than 1 network category; accordingly, counts in this panel reflect event-to-network assignments rather than unique events. This panel provides the hemisphere-resolved complement to the subclass-specific systems-level summaries shown in Figure 4C1. Abbreviations: AUD indicates auditory; CER, cerebellar; DMN, default mode; LIM, limbic; SAL, salience; SM, sensorimotor; STR, striatal; THAL, thalamic; and VIS, visual network category.

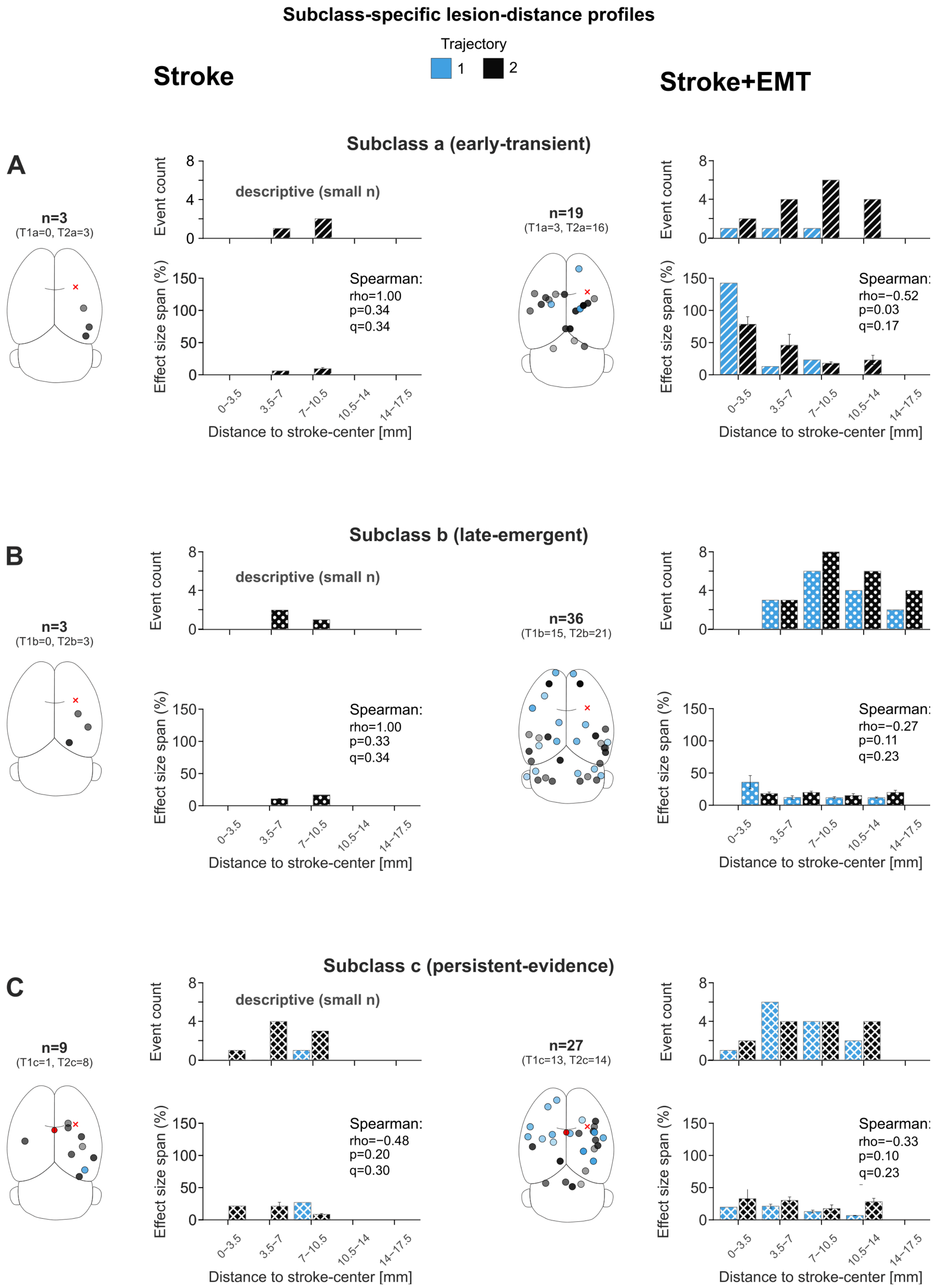

Figure S4. Subclass-specific spatial distribution and lesion-distance summaries of structural events. Structural events were stratified by timing-defined subclass and experimental group and displayed as spatial peak maps together with lesion-distance summaries. Rows correspond to (A) subclass a (early-transient), (B) subclass b (late-emergent), and (C) subclass c (persistent-evidence); columns correspond to Stroke and Stroke+EMT. Within each panel, the left subpanel shows the spatial distribution of event peaks relative to the lesion center (red cross), with colors indicating the 2 EWEP-derived main trajectories (T1 and T2). The upper-right subpanel shows the number of events per lesion-distance bin, and the lower-right subpanel shows the corresponding early-to-late effect-size span per bin, defined as the absolute difference between Week 1 and Week 8 effect-size values. Distances were grouped into 3.5-mm bins. Spearman correlation coefficients (ρ), nominal p values, and Benjamini-Hochberg-adjusted q values summarize the association between lesion distance and effect-size span within each subclass and group; significance testing was based on spatially blocked permutation tests. Total event counts are shown together with the distribution across the two main trajectories (T1, T2). Panels with very small event numbers are shown for descriptive purposes only.

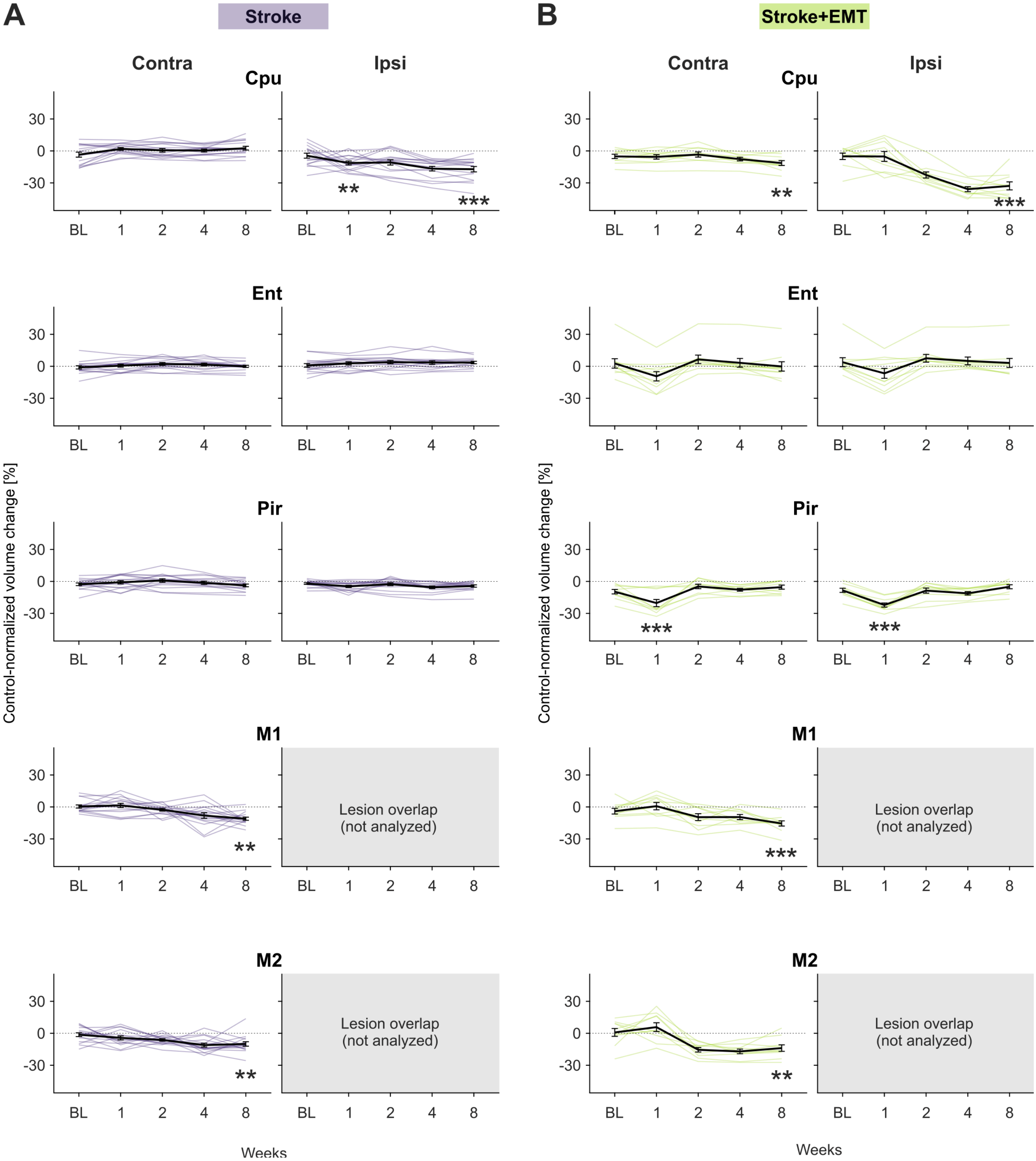

Figure S5. ROI-level longitudinal volume changes in selected regions after stroke and early multimodal training. Control-referenced ROI trajectories are shown for selected regions of interest (ROIs) in the Stroke and Stroke+EMT groups, separated by hemisphere. For each ROI, panels are arranged as contralateral and ipsilateral hemispheres for Stroke and Stroke+EMT. Thin colored lines represent individual animal trajectories, thick black lines represent group means, and error bars indicate SEM. Values are plotted as control-referenced percent volume change relative to the corresponding Control mean for the same ROI and hemisphere at each time point. Time points are baseline (BL) and follow-up at Weeks 1, 2, 4, and 8. Asterisks indicate intervention-versus-Control differences at Week 1 and Week 8, obtained from ROI- and hemisphere-specific linear mixed-effects models fit on raw ROI volumes with animal ID as a random intercept, followed by prespecified post hoc contrasts and Benjamini-Hochberg false discovery rate correction across ROI×Hemisphere tests within each contrast and time point; *q<0.05, **q<0.01, ***q<0.001. Panels labeled "Lesion overlap" were not analyzed because the ipsilateral motor ROI overlapped the lesion. Abbreviations: CPu indicates caudate-putamen; Ent, entorhinal cortex; Pir, piriform cortex; M1, primary motor cortex; and M2, secondary motor cortex.
